# The regulatory function of LexA is temperature-dependent in the deep-sea bacterium *Shewanella piezotolerans* WP3

**DOI:** 10.3389/fmicb.2015.00627

**Published:** 2015-06-18

**Authors:** Huahua Jian, Lei Xiong, Ying He, Xiang Xiao

**Affiliations:** ^1^State Key Laboratory of Microbial Metabolism, School of Life Sciences and Biotechnology, Shanghai Jiao Tong UniversityShanghai, China; ^2^State Key Laboratory of Ocean Engineering, School of Naval Architecture, Ocean and Civil Engineering, Shanghai Jiao Tong UniversityShanghai, China

**Keywords:** SOS response, LexA, microarray, temperature, deep-sea

## Abstract

The SOS response addresses DNA lesions and is conserved in the bacterial domain. The response is governed by the DNA binding protein LexA, which has been characterized in model microorganisms such as *Escherichia coli*. However, our understanding of its roles in deep-sea bacteria is limited. Here, the influence of LexA on the phenotype and gene transcription of *Shewanella piezotolerans* WP3 (WP3) was investigated by constructing a *lexA* deletion strain (WP3Δ*lexA*), which was compared with the wild-type strain. No growth defect was observed for WP3Δ*lexA*. A total of 481 and 108 genes were differentially expressed at 20 and 4°C, respectively, as demonstrated by comparative whole genome microarray analysis. Furthermore, the swarming motility and dimethylsulfoxide reduction assay demonstrated that the function of LexA was related to temperature. The transcription of the *lexA* gene was up-regulated during cold acclimatization and after cold shock, indicating that the higher expression level of LexA at low temperatures may be responsible for its temperature-dependent functions. The deep-sea microorganism *S. piezotolerans* WP3 is the only bacterial species whose SOS regulator has been demonstrated to be significantly influenced by environmental temperatures to date. Our data support the hypothesis that SOS is a formidable strategy used by bacteria against various environmental stresses.

## Introduction

Since it was first described by Radman (1975), the detailed pathway of the SOS response to DNA lesions had been thoroughly investigated in the model bacterium *Escherichia coli*. In addition to well-known classical DNA damaging agents such as UV-irradiation and antibiotics, several intracellular and extracellular processes and signals, including oxidative stress, acidic or alkaline stress, and high-pressure and acoustic cavitation, have also been revealed to be capable of triggering the SOS response (Erill et al., 2007). Stress-survival studies showed that an SOS-deficient mutant of *Listeria monocytogenes* was less resistant to heat (55°C), H_2_O_2_ and acid exposure (pH 3.4) compared to the wild-type cells (van der Veen et al., 2010). Additionally, the swarming motility was influenced by the SOS regulator RecA and conferred fitness to *Salmonella enterica* (Medina-Ruiz et al., 2010). Thus the SOS response has been recognized as an important strategy for bacterial survival and adaptation to changing environments. Furthermore, studies have demonstrated the crucial role of the SOS response in promoting the spread of mobile genetic elements (Beaber et al., 2003) and integron recombination, which are responsible for incorporating and expressing genes grouped as cassettes (Guerin et al., 2009). Thus, SOS also plays a considerable role in lateral gene transfer and evolution.

The LexA protein is a transcriptional regulator situated in the central position of the SOS pathway. The induction process of the SOS response has been well-described in several reviews (Michel, 2005; Kelley, 2006; Baharoglu and Mazel, 2014). Briefly, damage of the cellular DNA by external stressors produces single-stranded DNA (ssDNA); then, RecA bind to the ssDNA and becomes activated. The active RecA promotes the self-cleavage of the LexA dimer, releasing it from the promoter of the SOS regulon genes and derepressing their transcription. LexA homologues can be found in almost all sequenced bacterial genomes, suggesting both an ancient origin and widespread distribution of *lexA* and the SOS response (Erill et al., 2007). The LexA binding sites (SOS boxes) are rather conservative and have been used as a marker to indicate the evolutionary history of the SOS pathway in the bacterial domain (Abella et al., 2004; Mazón et al., 2004). Although genes of the LexA regulons are diverse in different bacteria and are not always equivalent to the SOS regulon (Kelley, 2006), there is no doubt that LexA is the key regulator in the SOS pathway (Butala et al., 2009). The regulatory function of LexA has been examined in various bacteria and was demonstrated to play important roles in growth, survival, hydrogenase expression, sporulation and antibiotic resistance (Tapias et al., 2000; Gutekunst et al., 2005; Rocha et al., 2008; Jochmann et al., 2009; Walter et al., 2014). However, the composition of the SOS regulon and the function of LexA in the deep-sea environment (which accounts for the majority of the ocean) is largely unknown. Considering the abyssal environment is characterized with permanent low temperatures (2–4°C), it would be interesting to testify whether bacteria which have evolved in a relatively constant habitat could be hyper-sensitive to temperature fluctuations, and thus systems associated with stress adaptation such as SOS pathway could manifest themselves differently depending on the thermal regime. Therefore, we focused on the investigation of the mechanism by which deficient SOS mutants of deep-sea bacterium respond to different temperatures in this study.

The *Shewanella* species are well-known for their versatile respiration ability and are widely distributed in aquatic environments, including the deep-sea. *Shewanella piezotolerans* WP3 (hereafter referred to as WP3) was previously isolated from a West Pacific sediment at a depth of 1914 m (Wang et al., 2004; Xiao et al., 2007). The temperature range of WP3 growth was characterized between 4 and 28°C at 0.1 MPa, with maximal growth at 20°C (Xiao et al., 2007). The cold adaptation mechanism has been investigated in WP3, and fatty acid biosynthesis and nitrate reductase were shown to respond to changes in temperature (Wang et al., 2009; Chen et al., 2011). In this study, the SOS regulon was characterized using whole genome microarray analysis of the *lexA* deletion mutant. The results indicated that the differentially expressed genes (DEGs) were significantly influenced by temperature. Further physiological testing confirmed the temperature-dependent function of LexA because the transcription of the *lexA* gene was up-regulated at 4°C and after cold-shock. To the best of our knowledge, this is the first report of the characterization of LexA in a deep-sea microorganism and the evaluation of the effect of temperature on LexA regulation. Taken together, our data implied an important role for LexA in the evolution of WP3 in response to the cold deep-sea environment.

## Materials and Methods

### Bacterial Strains, Culture Conditions and Growth Assay

All bacterial strains and plasmids used in this study are listed in **Table 1**. The *Shewanella* strains were cultured in 2216E marine medium (2216E; 5 g/l tryptone, 1 g/l yeast extract, 0.1 g/l FePO_4_, and 34 g/l NaCl) with shaking at 220 rpm at different temperatures under atmospheric pressure condition. *E. coli* strain WM3064 was incubated in lysogeny broth (LB) medium (10 g/l tryptone, 5 g/l yeast extract, and 10 g/l NaCl) at 37°C with the addition of 50 μg/ml of DL-α,𝜀-diaminopimelic acid (DAP). For solid medium, agar-A (Bio Basic Inc., Markham, ON, Canada) was added at a concentration of 1.5% (w/v). The antibiotics chloramphenicol (Cm) and kanamycin (Kan; Sigma, St. Louis, MO, USA) was added to the medium at concentrations of 12.5 and 50 μg/ml for the *Shewanella* strains, respectively. The anaerobic growth of the WP3 strain was determined using turbidity measurements at 600 nm with modified 2216E medium (Chen et al., 2011) supplemented with dimethylsulfoxide (DMSO; Sigma, St. Louis, MO, USA) as the sole electron acceptor.

**Table 1:** Bacterial strains and plasmids used in this study.

Stain or plasmids	Relevant genotype	Reference or source
***Escherichia coli* strain**	
WM3064	Donor strain for conjugation; Δ*dapA*	Gao et al. (2006)
***Shewanella piezotolerans* WP3 strains**	
WP3	Wild-type, GenBank accession number CP000472	Lab stock
WP3Δ*lexA*	WP3, deletion mutant of *lexA* gene, Kan^r^	This work
WP3Δ*lexA*-C	WP3, WP3Δ*lexA* with pSW2-*lexA*, Kan^r^ and Chl^r^	This work
**Plasmids**	
pRE112	Allelic-exchange vector; Cm^r^ *sacB*	Edwards et al. (1998)
pSW2	Shuttle vector for complementation; Chl^r^, derivative from filamentous bacteriophage SW1	Unpublished work
pRE112-*lexA*	pRE112 containing the PCR fragment for deleting *lexA* gene	This work
pSW2-*lexA*	pSW2 containing *lexA* and upstream promoter region	This work

### Construction of the *lexA* Gene Deletion Mutant and Complement Strain

A *lexA* deletion mutant was constructed using the previously described method (Wang et al., 2009). First, the upstream and downstream fragments flanking both sides of the *lexA* gene and a Kan resistance gene were amplified with the PCR primer pairs (**Table 2**). These three fragments were used as templates in a second fusion PCR, resulting in a fragment with a deletion in the *lexA* gene and insertion of the *kan^R^* gene. Then, the PCR product was cloned into pRE112 as a *Sac* I-*Kpn* I fragment, yielding pRE112-*lexA*. This plasmid was transformed into *E. coli* WM3064 and then into WP3 by two-parent conjugation. The transconjugant was selected by Cm and Kan resistance and verified by PCR. The WP3 strain with pRE112-*lexA* inserted into the chromosome was plated onto 2216E agar medium supplemented with Kan and 10% sucrose. A successful *lexA* deletion mutant was screened and confirmed by PCR. For complementation, the *Shewanella*- *E. coli* shuttle vector pSW2 was used as previously described (Chen et al., 2011). Briefly, the *lexA* gene with its native promoter region was amplified with the *pfu* DNA polymerase (Tiangen, Beijing, China). Both the PCR product and pSW2 were digested with *Sac* I and *Sph* I and ligated to generate pSW2-*lexA*. The recombinant plasmid was introduced into WM3064 and then into WP3Δ*lexA* by conjugation. The complementation strain was confirmed by PCR and enzyme digestion.

**Table 2:** Primers used in this study.

Primer name	Sequence (5′-3′)	Description
lexAUL	ATGAGCTCTCGCTTGCAGTGTTAGTCGCTTCT	*lexA* deletion
lexAUR	GCCAGTGCTTTTAAATGCTCTTCA	*lexA* deletion
lexAKU	ATTTAAAAGCACTGGCCCGGCGAACGTGGCGAGA	*lexA* deletion
lexAKD	GGCGGAACTCATAGAAGGCGGCGGTGGAATC	*lexA* deletion
lexADL	TTCTATGAGTTCCGCCCTTCTGCTGATTTTC	*lexA* deletion
lexADR	AAGGTACCTGCACTTTTGGCCACTAACTGTAA	*lexA* deletion
Chlfor	TAAATACCTGTGACGGAAGAT	Mutant confirmation
Chlrev	TATCACTTATTCAGGCGTAGC	Mutant confirmation
lexAcompFor	TTTTGAGCTCGATTCGCTTGCAGTGTTAGT	Mutant complementation
lexAcompRev	AATAGCATGCTCATTGCCAATCTCCATTGC	Mutant complementation
swp5118RTFor	AACAGCCAGCCGTAACGTT	qPCR
swp5118RTRev	GCACCATCTGCAGTTTGGA	qPCR
swp5124RTFor	TTTGGCCATCGAAGACATG	qPCR
swp5124RTRev	CCTTGCGGACTCGAGTAAC	qPCR
swpl499RTFor	TGCAAATGCTGACGCTGTATC	qPCR
swpl499RTRev	GATGGCGAGCACGGTAAGTT	qPCR
swp4822RTFor	CAAGGGCGCAATGAGCTAAC	qPCR
swp4822RTRev	GCCCCTGCTGACTCAATTGA	qPCR
swpl346RTFor	CTCGAACTGGACAATGAGTGTAT	qPCR
swpl346RTrev	GCACGAGAACGTCCATCAC	qPCR
swp0958RTFor	ATTGCAGCCAGTGATTTGG	qPCR
swp0958RTRev	AAGGGCGCTGATGGATCT	qPCR
swpl364RTFor	TTGCGTACCGCACGAGAA	qPCR
swpl364RTRev	TCCGCTGTCGGTTCATGAT	qPCR
swp0265RTFor	TGGCGAGCATGTCACTACAGA	qPCR
swp0265RTRev	GGGCTGATTTGCCATCCA	qPCR
swp3209RTFor	GGTGAGTTCAACGGCAAAGG	qPCR
swp3209RTRev	CGGTGTCATGGTACTCTTGTTTG	qPCR
swp3786RTFor	AATCAGGCCGACGTTGCA	qPCR
swp3786RTRev	CGGTAACGCTCGATATGCTTT	qPCR
pepNRTFor	TTAAGGCAATGGAAGCTGCAT	qPCR
pepNRTRev	CGTCTTTACCCGTTAATGATACGA	qPCR
recARTFor	GCAGCAGCGCAGAAGCA	qPCR
recARTRev	ATCCAAGGCATGTTCTGCAT	qPCR
lexARTFor	GGAACCGGAAGAGGTCGAA	qPCR
lexARTRev	GCTCACCTGCAGCAACTTGA	qPCR

### Cold Shock Assay, RNA Isolation and Real-Time qPCR

The cells used to assess the cold shock response were prepared as described previously (Jian et al., 2012). The samples were harvested by centrifugation and immediately placed in liquid nitrogen prior to RNA extraction. The WP3 strains were inoculated into 2216E medium at different temperatures as indicated in the text, and the culture was collected immediately when the cells reached the mid-exponential phase (OD_600_≈0.8). Total RNA extraction, reverse transcription and real-time quantitative real-time PCR (qPCR) were performed as described previously (Jian et al., 2013). The primer pairs (**Table 2**) used to amplify the selected genes in qPCR were designed using Primer Express software (Applied Biosystems, Foster City, CA, USA).

### Whole Genome Microarray Analysis

A microarray that contained 95% of the total predicted gene content of WP3 was designed and manufactured (CapitalBio, Beijing, China). The preparation of fluorescent dye-labeled DNA and hybridization image acquisition, data processing and clustering were performed as previously described (Jian et al., 2013). Briefly, the total RNA was reverse transcribed with SuperScript II (Invitrogen, Carlsbad, CA, USA), and the cDNAs were labeled with Cy3 and Cy5 using the Klenow enzyme (Takara Bio Inc., Japan) according to the manufacturers’ instructions. Labeled cDNA was purified with the PCR purification kit (Macherey-Nagel, Düren, Germany) and resuspended in elution buffer. The microarray slides were hybridized with cDNA prepared from 3 biological replicate samples. As a measure of technical replication, the dye-swap experiment was performed on each sample so that a total of six data points were available for every ORF on the microarray. A LuxScan 10K scanner and microarray scanner 2.3 software (CapitalBio, Beijing, China) were used for the array image acquisition. The linear normalization method was used for data analysis based on the expression levels of WP3 housekeeping genes in combination with the yeast external controls. The normalized data were log-transformed and loaded into MAANOVA under the R environment for multiple testing by fitting a mixed-effects ANOVA model (Wu et al., 2003). Microarray spots with P values <0.001 in the *F*-test were regarded as DEGs. All of the DEGs were confirmed with the Significance Analysis of Microarrays (SAM) software (Tusher et al., 2001).

### Swarming Motility Assay

For the motility assays, a 1 μl culture of each strain was placed on the swarming plates (2216E medium with 0.7% agar, Eiken Chemical, Tokyo, Japan). The plates were incubated at 20 and 4°C for 3 and 7 days, respectively. Motility was assessed by examining the migration distance of the bacteria from one side to another side of the colony edge (maximal swarming distance). For each strain, the assays were performed on at least three times with three independent cultures spotted onto three plates. The data were analyzed with Student’s two-tailed *t*-test using the Excel software (Microsoft Corporation, USA).

### Bioinformatic Analysis

The SOS Box was identified by searching the 5′ regions (1–700 bp upstream of the start codon) of the genes in WP3 using the conservative sequence of SOS box in γ-proteobacterium. The information matrix for the generation of the LexA Logo was produced by aligning the WP3 LexA binding sequences predicted by the RegPredict web server, which is available at http://regpredict.lbl.gov (Novichkov et al., 2010b). A graphical representation of the matrix through a Logo graph was obtained with Weblogo software, which is available at http://weblogo.berkeley.edu (Crooks et al., 2004).

## Results

### Construction of the *lexA* Deletion Mutant and Growth Assay

The intact *lexA* gene coding region was deleted from the WP3 genome, and the mutated strain was designated WP3Δ*lexA*. Initially, the putative impact of LexA on the growth of WP3 was investigated at different temperatures (Supplementary Figure S1). No growth deficiency of WP3Δ*lexA* was observed when the strain was cultivated either at 20°C (the optimal growth temperature of WP3) or 4°C (the *in situ* environmental temperature). Furthermore, although no significant filament phenotype was observed, the cell length of the *lexA* mutant was notably increased at 20°C, indicating that the *lexA* deletion had an effect on cell division and morphology (Supplementary Figure S2).

### Transcriptomic Profiling of WP3Δ*lexA* at Different Temperatures

**FIGURE 1:**
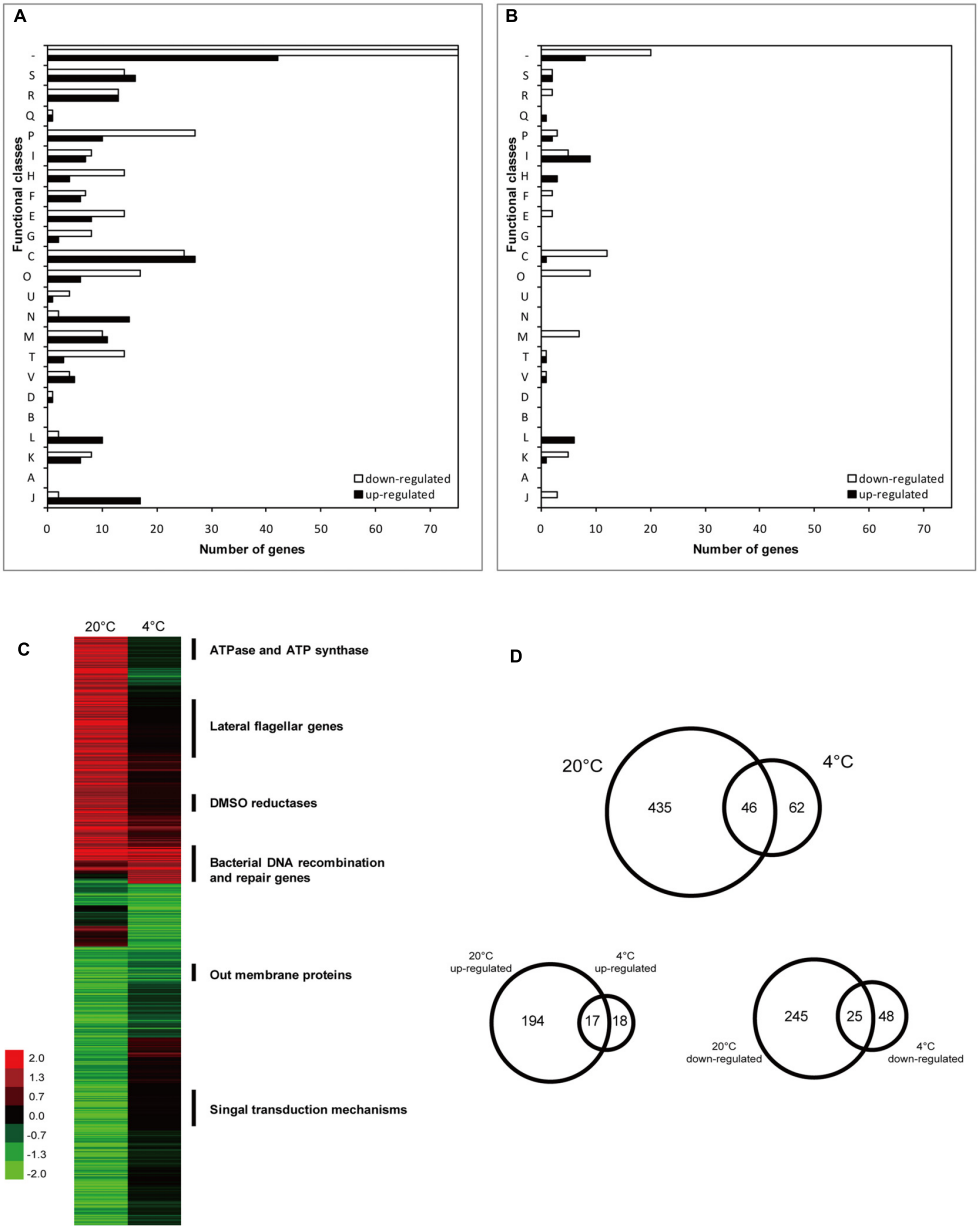
**Number and functional annotation of the differentially expressed genes (DEGs) according to their COG categories.** Bars indicate the number of genes in each group that showed significant changes in expression in WP3 after deletion of *lexA* gene at 20°C **(A)** and 4°C **(B)**, respectively. Genes were divided into functional categories according to NCBI (http://www.ncbi.nlm.nih.gov/COG/). Functional categories are abbreviated as follows: J, translation, ribosomal structure and biogenesis; A, RNA processing and modification; K, transcription; L, replication, recombination, and repair; B, chromatin structure and dynamics; D, cell cycle control, cell division, and chromosome partitioning; V, defense mechanisms; T, signal transduction mechanisms; M, cell wall/membrane/envelope biogenesis; N, cell motility; U, intracellular trafficking, secretion and vesicular transport; O, posttranslational modification, protein turnover, and chaperones; C, energy production and conversion; G, carbohydrate transport and metabolism; E, amino acid transport and metabolism; F, nucleotide transport and metabolism; H, coenzyme transport and metabolism; I, lipid transport and metabolism; P, inorganic ion transport and metabolism; Q, secondary-metabolite biosynthesis, transport, and catabolism; R, General function prediction only; S, Function unknown; and –, no COG identified. **(C)** Hierarchical clustering analysis of DEGs in WP3Δ*lexA*. The function of the enriched genes with different expression patterns at 20 and 4°C were indicated. Color legend is to the left, showing increased (green) and decreased (red) transcription levels. **(D)** Venn diagram displaying the numbers of DEGs at different temperatures.

Whole-genome microarray analysis was performed to identify possible LexA regulation targets by comparing the gene transcription profiles of WP3 and WP3Δ*lexA* at 20 and 4°C. Overall, 481 genes were found to be differentially expressed between these two strains at 20°C, including 211 up-regulated and 270 down-regulated genes (**Figure 1D**, Supplementary Tables S1 and S2). However, only 108 DEGs (35 and 73 genes with increased and decreased transcription levels, respectively) were detected in WP3Δ*lexA* at 4°C, indicating a temperature-dependent regulatory function for LexA in WP3.

To validate the microarray data, seven genes, including those that were up-regulated, down-regulated or unchanged, were selected for qPCR. It should be noted that the same samples were used for the microarray and qPCR. The relative mRNA levels for each gene were calculated and log-transformed. The correlation coefficients (*R*^2^) between the data obtained by microarray and qPCR were 0.948 and 0.899 at 20 and 4°C, respectively (Supplementary Figure S3), demonstrating that the microarray data were reliable and could be used for the follow-up analysis. The microarray data have been deposited in NCBI’s Gene Expression Omnibus (GEO) and are accessible through GEO series accession numbers GSE60724 and GSE60727.

Functional classification of differentially transcribed genes was performed using the Clusters of Orthologous Groups (COGs) of Proteins database (**Figures 1A,B**). Approximately 30.6 and 29.6% of the genes with altered expression levels at 20 and 4°C, respectively, had unknown functions or no COG identified. The enriched clusters at 20°C were associated with energy production and conversion (52/239, number of DEGs/total number of genes in the specific COG category), protein modification and turn over (23/162), inorganic ion transport and metabolism (37/172) and cell motility (17/134). However, only a few of the functional groups with high numbers of DEGs were identified at 4°C in WP3Δ*lexA*, including energy production and conversion (13/239) and lipid transport and metabolism (14/120).

Hierarchical clustering analysis was performed to investigate the effect of temperature on the regulatory function of LexA. This analysis demonstrated a significant difference in the expression patterns at 20 and 4°C (**Figure 1C**). More than 90% of the DEGs at 20°C did not show differential expression at 4°C, suggesting that LexA regulated more genes at higher temperatures. Interestingly, most of the common DEGs at the different temperatures showed the same tendency, including 17 induced and 25 reduced genes (**Figure 1D**).

The common up-regulated genes were annotated as DNA recombination and repair. These genes consisted of the core LexA regulon as reported in other bacteria (Abella et al., 2004; Erill et al., 2007), such as *recA* (bacterial DNA recombination protein), *recN* (DNA repair protein), *recG* (ATP-dependent DNA helicase), *dinP* (DNA damage inducible protein P), *topA* (DNA topoisomerase III) and *dnaE* (DNA polymerase III α subunit), indicating that the regulation of the core regulon by LexA was not affected by temperature. The functions of the common down-regulated gene were shown to be associated with the ribosomal subunit interface protein (swp3757 and swp3408), outer membrane protein (swp0247, swp0248, and swp3209), Acyl-CoA dehydrogenase (swp2312 and swp2982), fumarate reductase flavoprotein (swp0430) and twin-arginine translocation pathway signal (swp5029), suggesting that a similar strategy could exist in WP3 for coping with DNA damage at different temperatures.

### Temperature-Dependent Regulation of LexA in WP3

**FIGURE 2:**
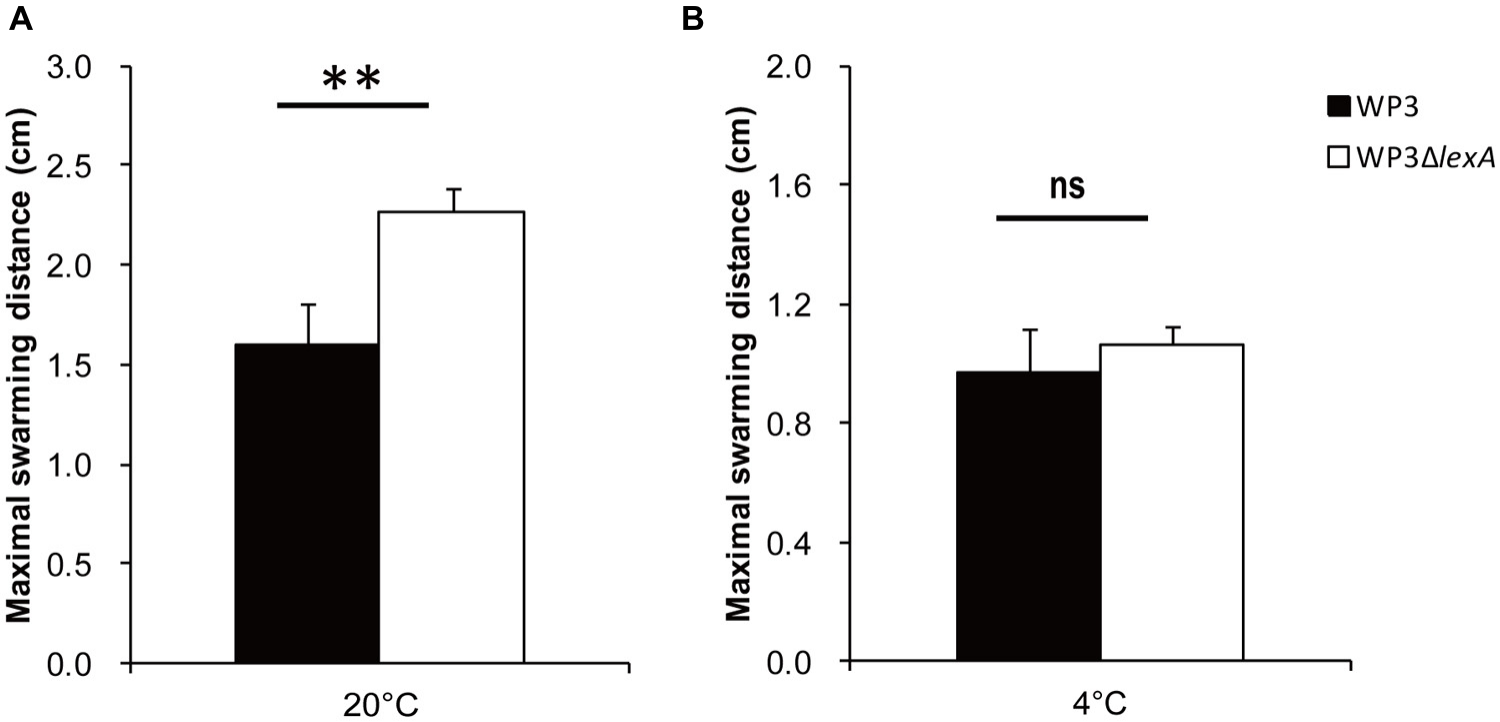
**The swarming motility assays of the *lexA* gene mutant at 20°C **(A)** and 4°C (B).** The data shown represent the results of two independent experiments, and the error bars indicate the SD. The data were analyzed by Student’s *t*-test, ^∗∗^*P* < 0.01; ns: not significantly different.

Because most of the DEGs exhibited different expression levels at 20°C, the characterization of these genes were of interest. Notably, a large portion of the lateral flagellar (*laf*) genes were up-regulated after *lexA* deletion at 20°C, but exhibited no significant difference in expression at 4°C (**Figure 1C**, Supplementary Tables S1 and S2). It is noteworthy that only one DEG was found in the polar flagellar gene cluster, indicating that LexA specifically regulated *laf* gene expression. To confirm this phenotypically, swarming motility assays were performed at different temperatures. The *lexA* mutant demonstrated a significantly higher (*p* < 0.01) swarming ability compared with the wild-type strain at 20°C, while no significant difference was detected at 4°C (**Figure 2**); this finding is consistent with the transcriptomic data.

**FIGURE 3:**
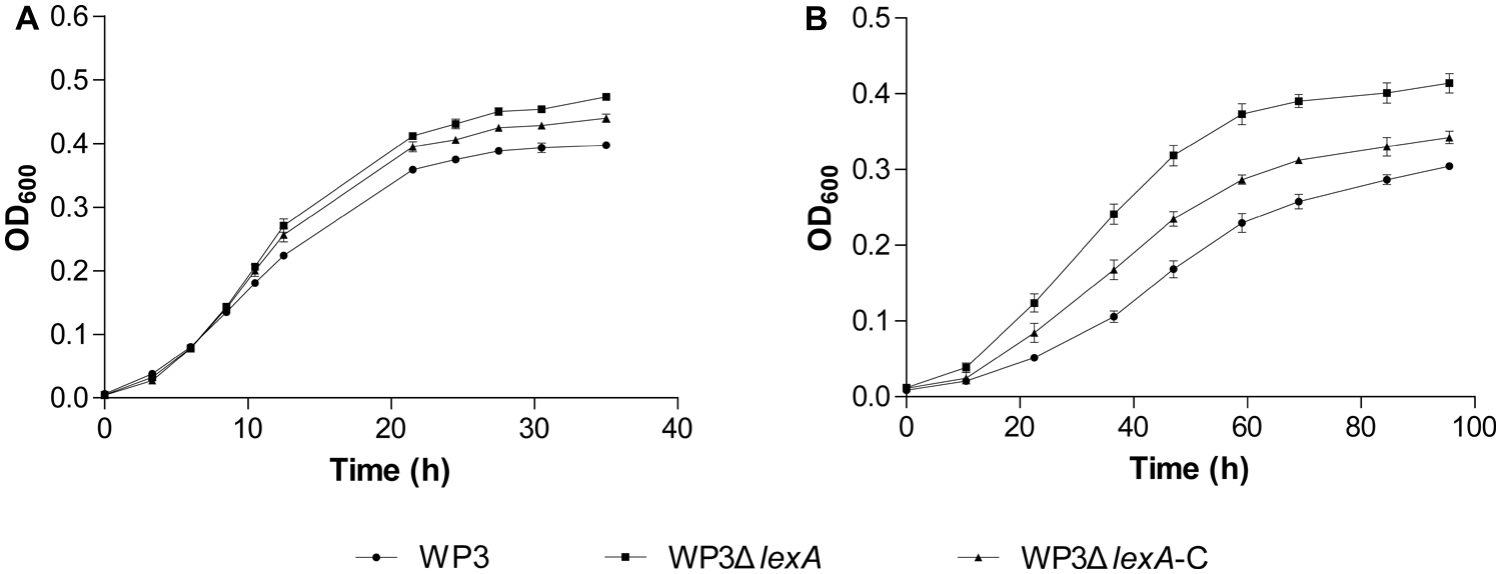
**Growth curve of WP3Δ*lexA* at 20°C **(A)** and 4°C **(B)** with DMSO as the sole electron acceptor.** All of the assays were performed in 2216E medium anaerobically supplemented with dimethylsulfoxide (DMSO). The average values and SD displayed by the error bars resulted from three replicates. All of the data shown represent at least two independent experiments.

Another similar case was the two DMSO reduction gene clusters (swp0721–swp0725 and swp3457–3459), which were significantly up-regulated at 20°C but not at 4°C. Surprisingly, the growth assay using DMSO as the sole electron acceptor indicated a more profound effect of the *lexA* mutation at 4°C (**Figure 3**), suggesting that a post-transcriptional effect may be involved in the LexA-regulated DMSO reduction process in WP3. Regardless, our data showed that LexA was involved in the regulation of anaerobic respiration of WP3 using DMSO in response to changes in temperature.

### The Transcription of the *lexA* Gene is Responsive to Cold Acclimatization and Cold Shock

**FIGURE 4:**
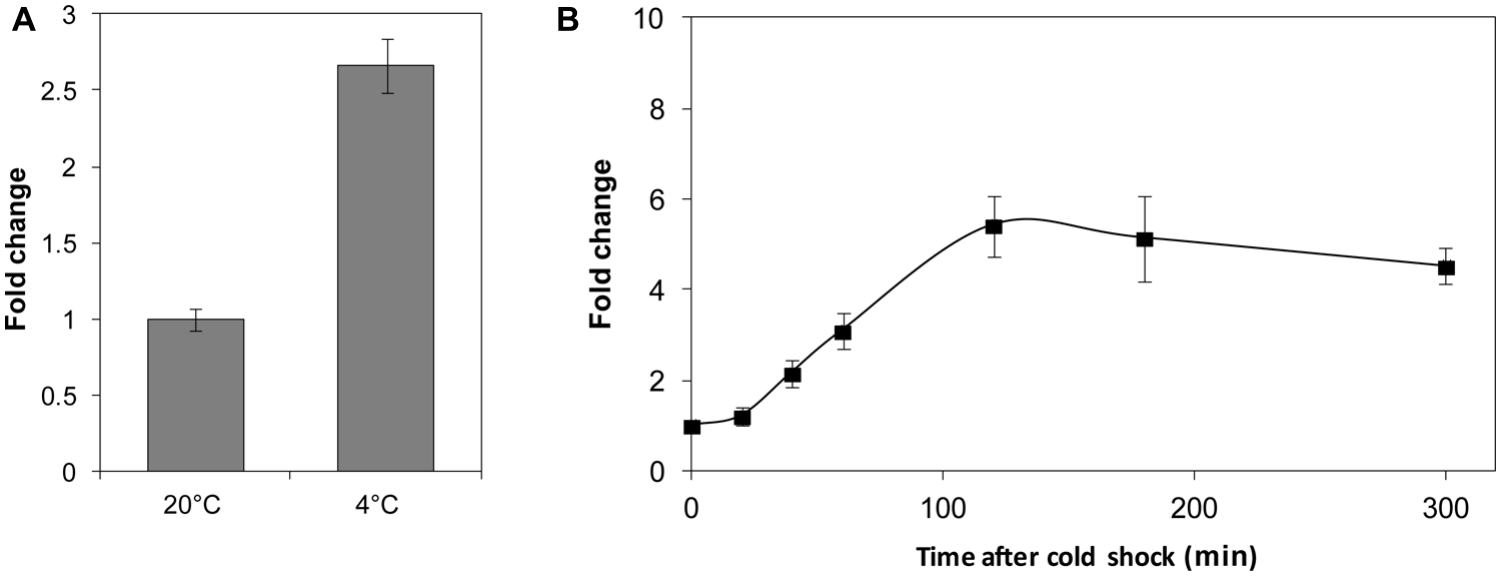
**The relative transcription level assay of the *lexA* gene at different temperatures **(A)** and after cold shock (B).** The transcription levels of WP3 at 20°C and before the 4°C cold shock were set as 1. The data shown represent two independent experiments, and the error bars indicate the SD.

As a transcriptional regulator, LexA plays a role as a DNA-binding protein. Thus, temperature may affect the LexA regulatory function in two ways: by influencing the quantity or the binding ability. First, the transcriptional level of the *lexA* gene was determined at different temperatures. A significantly higher mRNA (2.7-fold) level was observed at 4°C compared to 20°C (**Figure 4A**). We also investigated the transcription of the *lexA* gene in response to cold shock, and found a remarkable increase in the mRNA quantity of the *lexA* gene 2 h after the temperature shift from 20 to 4°C (**Figure 4B**).

**FIGURE 5:**
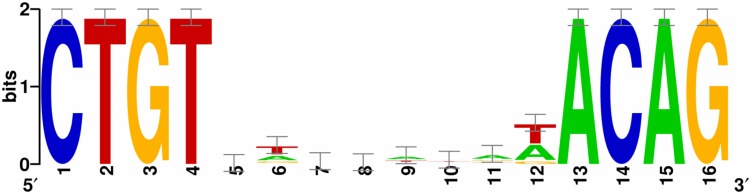
**The identification of the LexA binding box in *Shewanella piezotolerans* WP3.** A consensus LexA binding sequence logo was obtained from an unbiased search of the WP3 genome using the web-based tool RegPredict (http://regpredict.lbl.gov). The error bars indicate the SD of the sequence conservation.

Finally, we searched the WP3 genome for LexA binding sites using the conservative sequence of the SOS box in γ-proteobacterium (CTGTN_8_ACAG; Erill et al., 2003). The putative LexA binding sites located in the coding region were ruled out, and 20 SOS boxes were identified in the promoter region of 19 genes (**Table 3**). The highly conserved SOS box logo of WP3 is depicted (**Figure 5**); this sequence was found to be quite similar to other *Shewanella* stains (Erill et al., 2007; Novichkov et al., 2010a). Most of the genes with upstream LexA binding sites showed differential expression in WP3Δ*lexA*, but all showed similar changes at different temperatures. These results suggested that it was unlikely that temperature affected the binding of LexA to these target genes.

**Table 3:** LexA binding sites in the WP3 genome.

Gene ID	Gene	Gene products	Distance (bp) from ATG	Motif	Ratio (log_2_) WP3Δ*lexA*/WP3
Swp0246	–	Cation eﬄux protein	121	CTGTATTAGTAAACAG	–
Swp0839	–	Site-specific DNA-methyltransferase	58	CTGTTTTTATATACAG	1.01 (20°C)
Swpll79	*dinP*	DNA-damage-inducible protein P	93	CTGTTTTTATATACAG	2.18 (20°C) 1.57 (4°C)
Swp2821	*yebG*	DNA damage-inducible protein	28	CTGTTTTTATATACAG	2.41 (20°C) 1.72 (4°C)
Swp0958	*–*	CBS domain protein	63	CTGTGCGGTTTTACAG	-4.33 (20°C) -2.82 (4°C)
Swpl366	*recA*	bacterial DNA recombination protein	121	CTGTATGATTGTACAG	2.92 (20°C) 2.33 (4°C)
Swpl511	*flic*	Flagellin domain protein	128	CTGTTGATTAGTACAG	–
Swp2323	*–*	Conserved hypothetical protein	102	CTGTATGTATGTACAG	4.43 (20°C) 3.23 (4°C)
Swp2934	*fusA*	Translation elongation factor G	263	CTGTGACTATTTACAG	–
Swp4323	*–*	Conserved hypothetical protein	277	CTGTTAACTCCTACAG	-3.03 (20°C) -0.17 (4°C)
Swp4378	*–*	Hypothetical protein	136	CTGTCAACGCTAACAG	–
Swp4674	*–*	Macrolide-eﬄux protein	180	CTGTCGGTCGAGACAG	–
Swp4821	*lexA*	Transcriptional repressor	43	CTGTATATACTAACAG	-1.06 (20°C)
			24	CTGTATAGAAAAACAG	-1.37 (4°C)
Swpl346	*recN*	DNA repair protein	59	CTGTATAGAAAAACAG	2.77 (20°C)
					1.28 (4°C)
Swp5119	*lafB*	Flagellar hook-associated protein	96	CTGTAAAGTGCAACAG	–
Swp4746	*–*	Patatin	194	CTGTTATGTTATACAG	–
Swp3730	*ribF*	Riboflavin kinase / FAD synthetase	371	CTGTTTTGGCAAACAG	–
Swp2354	*ruvA*	DNA recombination protein	335	CTGTCATACCGGACAG	–
Swp0367	*romA*	Outer membrane protein	679	CTGTGGCCAGAAACAG	–

## Discussion

Previous studies showed that the *lexA* gene deletion was lethal for *E. coli* and other γ-proteobacteria, such as *Pseudomonas putida*, *P. aeruginosa, Aeromonas hydrophila*, *Erwinia carotovora*, and *Salmonella typhimurium* (Walker, 1984; Calero et al., 1993; Riera and Barbe, 1995). Therefore, viable *E. coli lexA*-defective strains must be constructed based on the mutation of the *sfiA* gene (Walker, 1984). In this study, the *lexA* deletion mutant of deep-sea bacterium WP3 was constructed directly; no growth deficiency was observed in the growth assay (Supplementary Figure S1), which is different from the decreased growth rate of *lexA* mutants reported for other bacteria (Rocha et al., 2008; Jochmann et al., 2009). However, there is also a report indicating that the growth and viability of *Rhodobacter sphaeroides lexA* (Def) cultures do not show any change relative to the wild-type strain (Tapias et al., 2000). Therefore, the deletion of *lexA* leading to the defect of cell growth should not be considered as a rule within the bacterial domain. No SOS boxes were found in the promoter region of the *sulA* gene, which encodes the cell division suppressor (Adams and Errington, 2009). Moreover, no increased transcription of *sulA* was observed, indicating that this gene does not belong to the SOS regulon in WP3. Additionally, the cell length of WP3Δ*lexA* was increased significantly at 20°C but not at 4°C (Supplementary Figure S2), suggesting a profound influence of the *lexA* deletion at higher temperatures. In term of this phenotype, the posttranscriptional regulation of *sulA* gene expression or alternative cell division regulators could be responsible. The loss of control of cell division and reproduction by LexA in WP3 may be due to one of the adaptive strategies developed for survival in the deep-sea sediment, which is characterized by aphotic and relatively stable environmental factors. Thus the DNA lesions were speculated to be too low to necessitate LexA control of cell division inhibition in the bathypelagic microorganism.

The binding sites of LexA in WP3 were analyzed and the conservative sequence was demonstrated to share high similarity with other *Shewanella* strains, indicating an evolutionary history of LexA in this widespread genus. Interestingly, the genes harboring LexA boxes did not show differential expression at 20 and 4°C (**Table 2**), implying that the transcription of these genes regulated by LexA was not affected by temperature. Because our qPCR results indicated that the transcriptional level of the *lexA* gene increased during cold acclimation and after cold shock, the temperature-dependent regulation of LexA may be due to its different concentrations at various temperatures. Indeed, the intracellular level of LexA in *E. coli* varied markedly throughout the *E. coli* growth cycle (Dri and Moreau, 1993).

In this study, the LexA-regulated genes were demonstrated to respond to the change in temperature, with the number of DEGs at 4°C accounting for less than 1/4 of the DEGs at 20°C (**Figure 1**). The LexA regulon of *E. coli* was reported to be controlled by pH, and shifts of the bacterial cell to extreme pH increased the cellular concentration of the LexA repressor (Dri and Moreau, 1994). The growth of a LexA-depleted *Synechococcus* strain was found to be strongly dependent on the availability of inorganic carbon (Domain et al., 2004). Interestingly, a physiological test confirmed the temperature-dependent function of LexA in swarming motility and DMSO reduction (**Figures 2** and **3**). The analysis of gene expression profile in *lexA* gene mutant at 20 MPa and 4°C also demonstrated that the transcription of one DMSO reduction gene cluster (swp0721–swp0725) was significantly changed (Jian and Wang, 2015). Considering that the deep-sea is generally an oligotrophic environment with limited energy resources (Orcutt et al., 2011), LexA may be involved in the adjusting of the life-strategy and adaptation of WP3 to deep-sea sediments by regulation of anaerobic respiration such as DMSO reduction.

In addition to modulate the SOS response by preventing SulA inhibition of cell division in *E. coli*, it was revealed that the ATP-dependent protease LonS, regulates swarmer cell differentiation of *Vibrio parahaemolyticus* (Stewart et al., 1997). However, the SulA was demonstrated not responsible for *laf* gene expression (Gode-Potratz et al., 2011). As SOS boxes were identified both in the promoter region of polar (*fliC*) and *laf* gene (*lafB*), the LonS and LexA were speculated to play a role in the regulation of flagella synthesis and motility. Moreover, the thermo-regulation of swarming motility and *laf* gene has been reported in several bacteria (Ulitzur, 1975; Bresolin et al., 2008; Soo et al., 2008; Tambalo et al., 2010; Hockett et al., 2013). In this study, the increasing of swarming motility in WP3Δ*lexA* was only observed at 20°C. Furthermore, the transcriptional level of *laf* genes were up-regulated at 4°C according to our previous study (Wang et al., 2008). In conclusion, our data support the notion that *laf* system is an inducible system, and it is regulated by environmental factors including SOS response (Merino et al., 2006; Eloe et al., 2008; Lauro and Bartlett, 2008).

The comparative analysis of gene expression profile in *lexA* mutant at different temperature and pressures has been performed, and 19 DEGs were identified under all of the tested conditions (Supplementary Figure S4). These genes are mainly belonging to core SOS regulon (*recA, recN, dinP, dinG, dnaE, and TopA*). However, the majority of DEGs (206 genes) were shown to be specific at low-temperature and high-pressure, indicating the regulatory function of LexA is remarkablely influenced by environmental factors. Thus the flexible LexA regulon and conserved core genes and binding sites indicate the evolution of the SOS network during the adaptation process. Taken together, our data renewed the understanding of the role of LexA in sensing and adaptation to different environmental conditions. This finding will certainly stimulate the analysis of the SOS system to address global stress in bathypelagic ecosystems.

## Conflict of Interest Statement

The authors declare that the research was conducted in the absence of any commercial or financial relationships that could be construed as a potential conflict of interest.
