# Supplementary material for: The regulatory function of LexA is temperature-dependent in the deep-sea bacterium Shewanella piezotolerans WP3
Source: Front Microbiol. 2015 Jun 18;6:627. doi: 10.3389/fmicb.2015.00627 (PMC4471891; doi:10.3389/fmicb.2015.00627)
Supplement: Supplementary file 1 [file Data_Sheet_1.DOC]

***Supplementary Material***

**The regulatory function of LexA is temperature-dependent in the deep-sea bacterium *Shewanella piezotolerans* WP3**

**Huahua Jian^1^, Lei Xiong^1^, Ying He^1^, Xiang Xiao^1,2^***

^1^State Key Laboratory of Microbial Metabolism, School of Life Sciences and Biotechnology, Shanghai Jiao Tong University, Shanghai, PR China

^2^ State Key Laboratory of Ocean Engineering, School of Naval Architecture, Ocean and Civil Engineering, Shanghai Jiao Tong University, Shanghai, PR China

*** Correspondence:** Xiang Xiao, State Key Laboratory of Microbial Metabolism, School of Life Sciences and Biotechnology, Shanghai Jiao Tong University, No. 800 Dongchuan Road, Shanghai, 200240, PR China

[xoxiang@sjtu.edu.cn](mailto:xoxiang@sjtu.edu.cn)

**Figures**

**Figure S1.** Growth curve of WP3Δ*lexA* at 20°C (A) and 4°C (B). All of the assays were performed in 2216E medium. The average values and standard deviation displayed by error bars resulted from three replicates. All of the data shown represent at least two independent experiments.

**Figure S2.** Effect of *lexA* gene mutation on the morphology of WP3 cells at 20°C and 4°C. The black line bar represents 10 μm.

**Figure S3.** Correlation analysis of the microarray and real-time qPCR assays. A total of 7 genes showing differential expression levels were randomly selected for this assay. The real time qPCR log_2_ values were plotted against the microarray log_2_ values.

**Figure S4.** Comparison of gene expression profile in WP3Δ*lexA* under different conditions. (A) Hierarchical clustering analysis of differentially expressed genes in WP3Δ*lexA*. The function of the enriched genes with different expression patterns were indicated. Color legend is to the left, showing increased (green) and decreased (red) transcription levels. (B) Venn diagram displaying the numbers of differentially expressed genes at different temperatures and pressures.
