# Supplementary figures and images for: The regulatory function of LexA is temperature-dependent in the deep-sea bacterium Shewanella piezotolerans WP3
Source: Front Microbiol. 2015 Jun 18;6:627. doi: 10.3389/fmicb.2015.00627 (PMC4471891; doi:10.3389/fmicb.2015.00627)

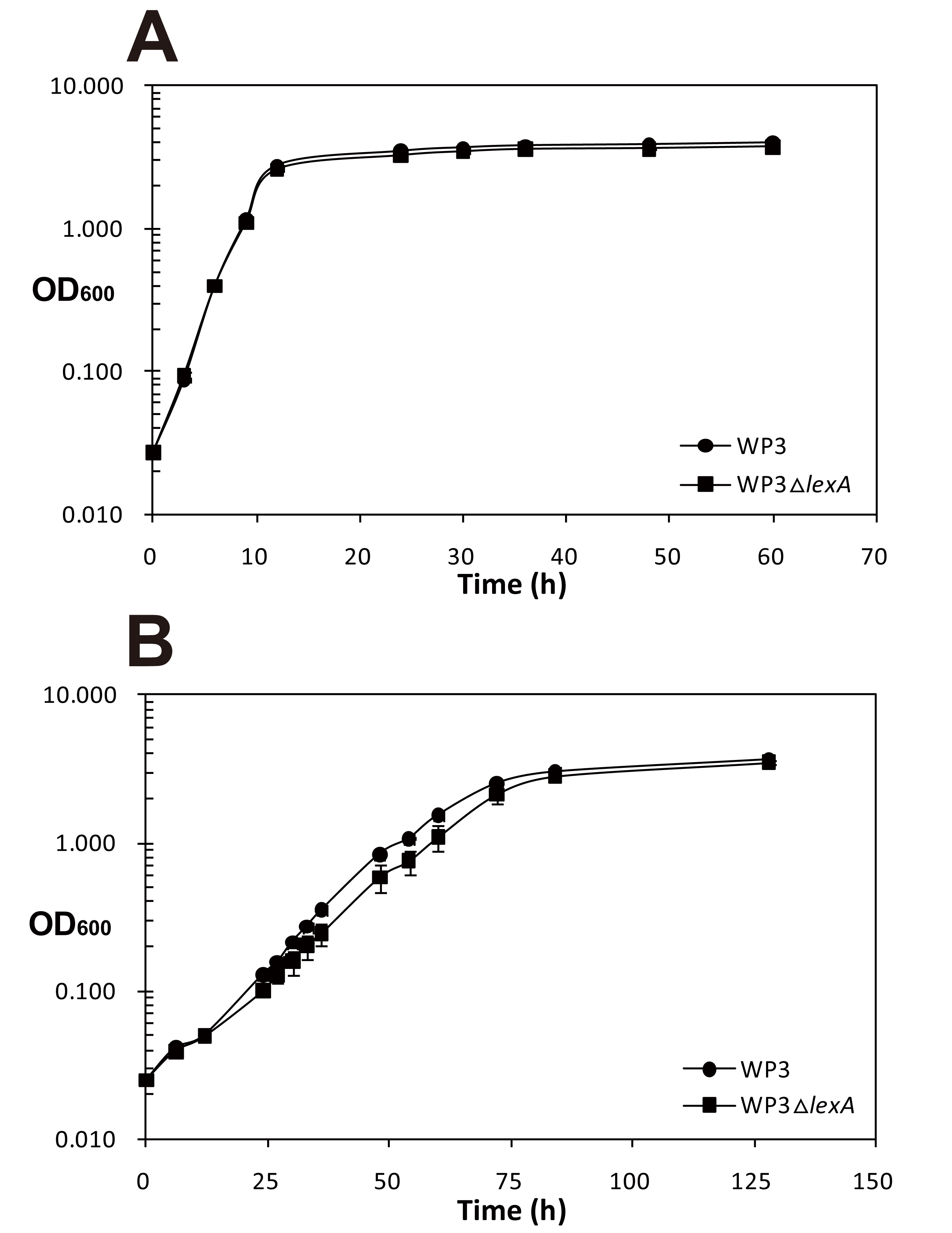

Supplement: Supplementary file 2 [file Image_1.TIF]

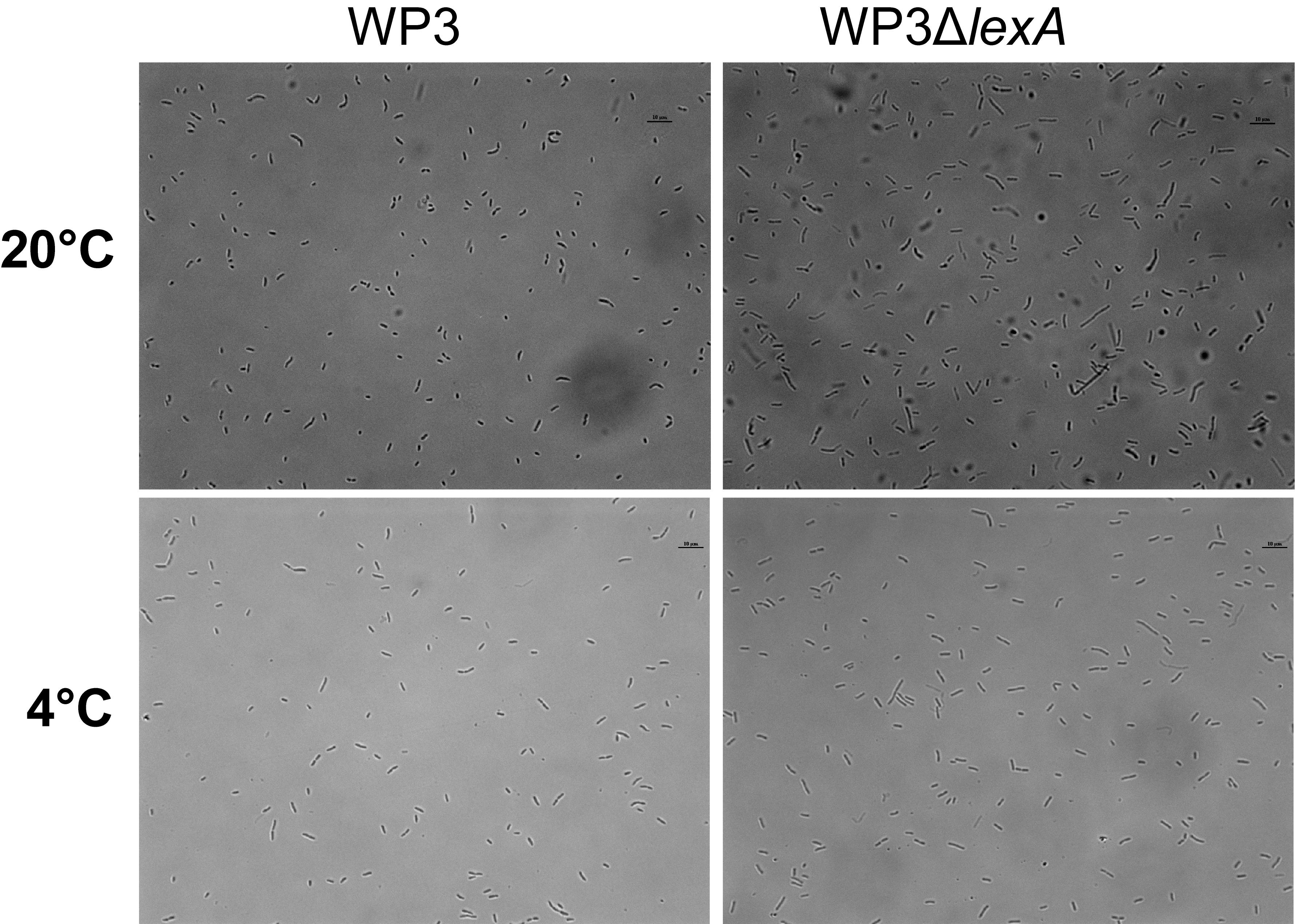

Supplement: Supplementary file 3 [file Image_2.TIF]

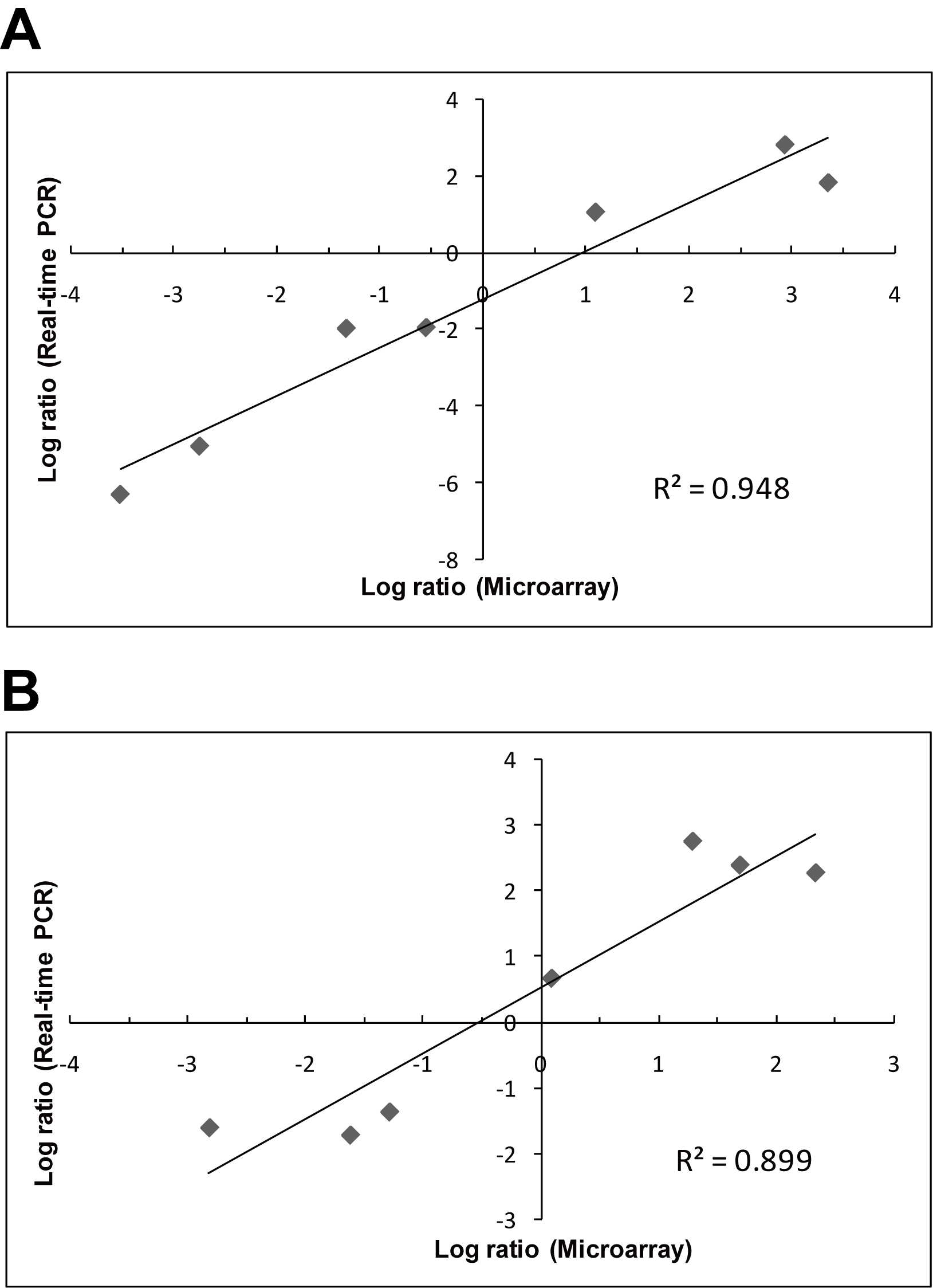

Supplement: Supplementary file 4 [file Image_3.TIF]

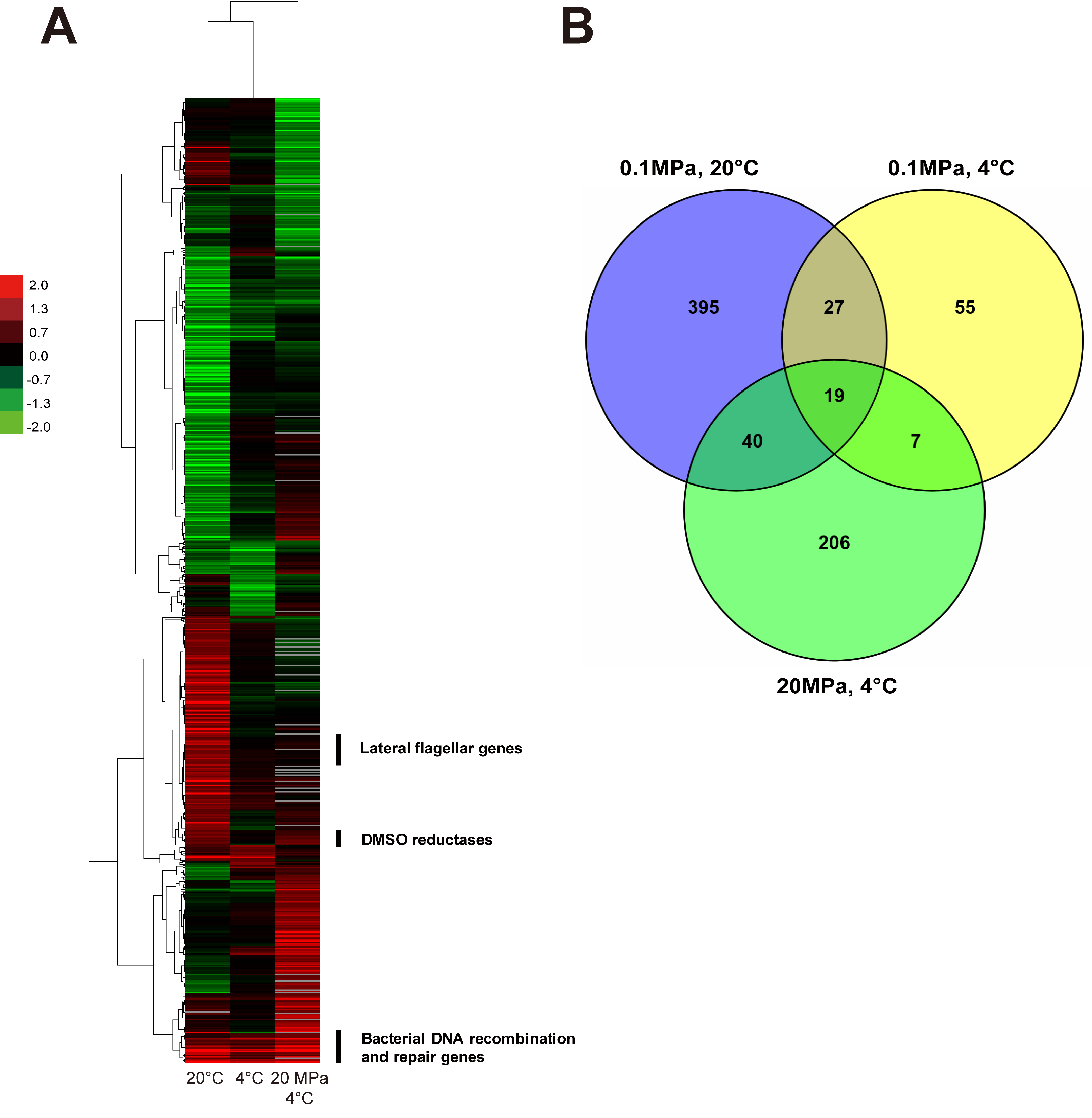

Supplement: Supplementary file 5 [file Image_4.TIF]
