# Supplementary material for: The regulatory function of LexA is temperature-dependent in the deep-sea bacterium Shewanella piezotolerans WP3
Source: Front Microbiol. 2015 Jun 18;6:627. doi: 10.3389/fmicb.2015.00627 (PMC4471891; doi:10.3389/fmicb.2015.00627)
Supplement: Supplementary file 6 [file Table_1.DOC]

**Table S1. Differentially expressed genes in WP3Δ*lexA* at 20°C.**

| **Functional category and gene locus designation** | **Avg fold change (WP3Δ*lexA* /WP3)** | | | **Product and description** | | | | | | | | | | | **COG designation or**  **no. of relevant genes/**  **total no. of genes** | | |  | |
| --- | --- | --- | --- | --- | --- | --- | --- | --- | --- | --- | --- | --- | --- | --- | --- | --- | --- | --- | --- |
| **Information storage and processing** | | | | | | | | | | | | | | | **45/580** | | |  | |
| Translation, ribosomal structure and biogenesis [J] | | | | | | | | | | | | | | | 19/177 | | |  | |
| swp3757 | 0.204 | | | Ribosomal subunit interface protein | | | | | | | | | | | COG1544 | | |  | |
| swp3408 | 0.468 | | | Ribosomal subunit interface protein | | | | | | | | | | | COG1544 | | |  | |
| swp3521 | 2.001 | | | Elongation factor Ts | | | | | | | | | | | COG0264 | | |  | |
| swp2587 | 2.018 | | | Seryl-tRNA synthetase, class IIa | | | | | | | | | | | COG0172 | | |  | |
| swp3733 | 2.035 | | | Ribosomal protein S20 | | | | | | | | | | | COG0268 | | |  | |
| swp2792 | 2.036 | | | Ribosomal protein L25 | | | | | | | | | | | COG1825 | | |  | |
| swp2026 | 2.058 | | | Ribosomal protein L6 | | | | | | | | | | | COG0097 | | |  | |
| swp2007 | 2.107 | | | Translation elongation factor G:Small GTP-binding protein domain | | | | | | | | | | | COG0480 | | |  | |
| swp2204 | 2.285 | | | Ribosomal protein L28 | | | | | | | | | | | COG0227 | | |  | |
| swp0905 | 2.374 | | | Valyl-tRNA synthetase, class Ia | | | | | | | | | | | COG0525 | | |  | |
| swp4303 | 2.401 | | | Lysyl-tRNA synthetase, class-2 | | | | | | | | | | | COG1190 | | |  | |
| swp0702 | 2.422 | | | Ribosomal protein S9 | | | | | | | | | | | COG0103 | | |  | |
| swp1367 | 2.440 | | | Alanyl-tRNA synthetase | | | | | | | | | | | COG0013 | | |  | |
| swp3914 | 2.490 | | | Leucyl-tRNA synthetase bacterial/mitochondrial, class Ia | | | | | | | | | | | COG0495 | | |  | |
| swp0477 | 2.505 | | | Ribosomal protein L31 | | | | | | | | | | | COG0254 | | |  | |
| swp1219 | 2.509 | | | Ribosome-binding factor A | | | | | | | | | | | COG0858 | | |  | |
| swp0020 | 2.818 | | | Glycyl-tRNA synthetase, beta subunit | | | | | | | | | | | COG0751 | | |  | |
| swp1589 | 3.639 | | | Sulfate adenylate transferase, subunit 1 | | | | | | | | | | | COG0050 | | |  | |
| swp3056 | 3.652 | | | Ribonuclease E | | | | | | | | | | | COG1530 | | |  | |
| Transcription [K] | | | | | | | | | | | | | | | 13/219 | | |  | |
| swp0723 | 0.089 | | | Transcriptional regulator, LysR family | | | | | | | | | | | COG0583 | | |  | |
| swp0842 | 0.251 | | | Regulatory protein, TetR | | | | | | | | | | | COG1309 | | |  | |
| swp4975 | 0.286 | | | GCN5-related N-acetyltransferase | | | | | | | | | | | COG0454 | | |  | |
| swp4465 | 0.316 | | | Regulatory protein, LysR:LysR, substrate-binding | | | | | | | | | | | COG0583 | | |  | |
| swp0050 | 0.366 | | | Regulatory protein, ArsR | | | | | | | | | | | COG0640 | | |  | |
| swp1854 | 0.459 | | | Transcriptional regulator, MarR family | | | | | | | | | | | COG1846 | | |  | |
| swp1064 | 0.462 | | | Transcriptional regulator | | | | | | | | | | | COG4977 | | |  | |
| swp5124 | 2.121 | | | FliA, Flagellar biosynthesis sigma-28 factor | | | | | | | | | | | COG1191 | | |  | |
| swp2036 | 2.272 | | | DNA-directed RNA polymerase | | | | | | | | | | | COG0202 | | |  | |
| swp3212 | 2.370 | | | Helicase c2 | | | | | | | | | | | COG1199 | | |  | |
| swp0132 | 2.499 | | | Histidine utilization repressor | | | | | | | | | | | COG2188 | | |  | |
| swp3949 | 2.765 | | | Penicillinase repressor | | | | | | | | | | | COG3682 | | |  | |
| swp0425 | 3.082 | | | Helix-turn-helix, Fis-type | | | | | | | | | | | COG2901 | | |  | |
| Replication, recombination and repair [L] | | | | | | | | | | | | | | | 12/182 | | |  | |
| swp3668 | 0.364 | | | | | | 5'-3' exonuclease | | | | | | | | COG0258 | | |  | |
| swp1664 | 0.478 | | | | | | Methylated-DNA-[protein]-cysteine S-methyltransferase | | | | | | | | COG0350 | | |  | |
| swp0839 | 2.012 | | | | | | Site-specific DNA-methyltransferase | | | | | | | | COG0270 | | |  | |
| swp1755 | 2.038 | | | | | | Transcription-repair coupling factor | | | | | | | | COG1197 | | |  | |
| swp0309 | 2.602 | | | | | | ATP-dependent DNA helicase RecG | | | | | | | | COG1200 | | |  | |
| swp2776 | 2.735 | | | | | | ATP-dependent helicase HrpA | | | | | | | | COG1643 | | |  | |
| swp1179 | 4.522 | | | | | | DNA-directed DNA polymerase | | | | | | | | COG0389 | | |  | |
| swp3111 | 5.070 | | | | | | DNA topoisomerase III | | | | | | | | COG0550 | | |  | |
| swp1346 | 6.803 | | | | | | DNA repair protein RecN | | | | | | | | COG0497 | | |  | |
| swp1366 | 7.591 | | | | | | RecA bacterial DNA recombination protein | | | | | | | | COG0468 | | |  | |
| swp2325 | 8.807 | | | | | | DNA polymerase III alpha subunit | | | | | | | | COG0587 | | |  | |
| swp2324 | 22.828 | | | | | | Nucleotidyltransferase/DNA polymerase involved in DNA repair | | | | | | | | COG0389 | | |  | |
| **Cellular processes and signaling** | | | | | | | | | | | | | | | **94/827** | | |  | |
| Cell cycle control, cell division, chromosome partitioning [D] | | | | | | | | | | | | | | | 2/30 | | |  | |
| swp4822 | 0.148 | | | | SOS-response cell division inhibitor, blocks FtsZ ring formation | | | | | | | | | | COG5404 | | |  | |
| swp0473 | 2.316 | | | | Sporulation related | | | | | | | | | | COG3087 | | |  | |
| Defense mechanisms [V] | | | | | | | | | | | | | | | 9/79 | | |  | |
| swp4754 | 0.351 | | | AmpE protein | | | | | | | | | | | COG3725 | | |  | |
| swp1329 | 0.414 | | | Acriflavin resistance protein | | | | | | | | | | | COG0841 | | |  | |
| swp0629 | 0.450 | | | Multidrug resistance efflux pump | | | | | | | | | | | COG1566 | | |  | |
| swp0805 | 0.450 | | | CydD, ABC transporter, ATP-binding protein CydD | | | | | | | | | | | COG1132 | | |  | |
| swp1784 | 2.093 | | | Acriflavin resistance plasma membrane protein | | | | | | | | | | | COG0841 | | |  | |
| swp2732 | 2.170 | | | Acriflavin resistance protein | | | | | | | | | | | COG0841 | | |  | |
| swp0577 | 2.310 | | | Acriflavin resistance protein | | | | | | | | | | | COG0841 | | |  | |
| swp2547 | 4.745 | | | Acriflavin resistance protein | | | | | | | | | | | COG0841 | | |  | |
| swp2969 | 5.042 | | | AcrB/AcrD/AcrF family protein | | | | | | | | | | | COG0841 | | |  | |
| Signal transduction mechanisms [T] | | | | | | | | | | | | | | | 17/159 | | |  | |
| swp0692 | 0.034 | | | Response regulator receiver | | | | | | | | | | | COG0745 | | |  | |
| swp1055 | 0.087 | | | UspA (Universal stress protein) | | | | | | | | | | | COG0589 | | |  | |
| swp4459 | 0.090 | | | UspA (Universal stress protein) | | | | | | | | | | | COG0589 | | |  | |
| swp1057 | 0.129 | | | UspA (Universal stress protein) | | | | | | | | | | | COG0589 | | |  | |
| swp0567 | 0.258 | | | PhoH-like protein | | | | | | | | | | | COG1875 | | |  | |
| swp3960 | 0.311 | | | ATPase-like:Histidine kinase | | | | | | | | | | | COG3850 | | |  | |
| swp4354 | 0.317 | | | Helix-turn-helix, Fis-type | | | | | | | | | | | COG2204 | | |  | |
| swp0881 | 0.319 | | | Response regulator receiver:Transcriptional regulatory protein, C-terminal | | | | | | | | | | | COG0745 | | |  | |
| swp1518 | 0.321 | | | Helix-turn-helix, Fis-type | | | | | | | | | | | COG2204 | | |  | |
| swp3783 | 0.407 | | | Sigma-E factor regulatory protein RseC | | | | | | | | | | | COG3086 | | |  | |
| swp2415 | 0.425 | | | Response regulator | | | | | | | | | | | COG2197 | | |  | |
| swp2789 | 0.473 | | | PrkA serine kinase | | | | | | | | | | | COG2766 | | |  | |
| swp0311 | 0.480 | | | Sensor histidine kinase, putative | | | | | | | | | | | COG4585 | | |  | |
| swp3784 | 0.497 | | | Anti-sigmaE factor RseB | | | | | | | | | | | COG3026 | | |  | |
| swp0019 | 2.383 | | | Signal transduction protein, putative | | | | | | | | | | | COG1639 | | |  | |
| swp1573 | 2.811 | | | Cytoplasmic phosphatase | | | | | | | | | | | COG0394 | | |  | |
| swp2820 | 3.203 | | | Putative GAF sensor protein | | | | | | | | | | | COG1956 | | |  | |
| Cell wall/membrane/envelope biogenesis [M] | | | | | | | | | | | | | | | 21/214 | | |  | |
| swp3209 | 0.074 | | | | | | Outer membrane porin, putative | | | | | | | | COG3203 | | |  | |
| swp3395 | 0.170 | | | | | | OmpW, outer membrane protein W | | | | | | | | COG3047 | | |  | |
| swp1330 | 0.186 | | | | | | HlyD family secretion protein | | | | | | | | COG0845 | | |  | |
| swp4044 | 0.215 | | | | | | Glycosyl transferase, family 4 | | | | | | | | COG0438 | | |  | |
| swp3176 | 0.421 | | | | | | Peptidase M23B | | | | | | | | COG0739 | | |  | |
| swp2699 | 0.431 | | | | | | Peptidase M15B and M15C, D,D-carboxypeptidase VanY/endolysins | | | | | | | | COG1876 | | |  | |
| swp1971 | 0.453 | | | | | | Enzyme of sugar metabolism, putative | | | | | | | | COG0451 | | |  | |
| swp4021 | 0.469 | | | | | | TonB, C-terminal | | | | | | | | COG0810 | | |  | |
| swp0248 | 0.479 | | | | | | OmpA-like transmembrane region | | | | | | | | COG3637 | | |  | |
| swp0247 | 0.481 | | | | | | Outer membrane protein, putative | | | | | | | | COG3637 | | |  | |
| swp3727 | 2.347 | | | | | | Peptidase A8, signal peptidase II | | | | | | | | COG0597 | | |  | |
| swp1580 | 2.358 | | | | | | Glycosyl transferase, family 4 | | | | | | | | COG0438 | | |  | |
| swp4232 | 2.396 | | | | | | HlyD family secretion protein | | | | | | | | COG0845 | | |  | |
| swp4846 | 2.785 | | | | | | Sulfatase | | | | | | | | COG1368 | | |  | |
| swp3513 | 2.844 | | | | | | Bacterial surface antigen | | | | | | | | COG4775 | | |  | |
| swp2733 | 2.965 | | | | | | HlyD family secretion protein | | | | | | | | COG0845 | | |  | |
| swp2548 | 3.600 | | | | | | HlyD family secretion protein | | | | | | | | COG0845 | | |  | |
| swp1572 | 3.674 | | | | | | Periplasmic protein involved in capsular polysaccharide export | | | | | | | | COG1596 | | |  | |
| swp1586 | 3.910 | | | | | | Pyridoxal phosphate-dependent enzyme, putative | | | | | | | | COG0399 | | |  | |
| swp0270 | 4.280 | | | | | | Small GTP-binding protein domain:GTP-binding protein TypA | | | | | | | | COG0481 | | |  | |
| swp0383 | 7.152 | | | | | | MltA-interacting MipA | | | | | | | | COG3713 | | |  | |
| Cell motility [N] | | | | | | | | | | | | | | | 17/134 | | |  | |
| swp1896 | | | 0.296 | | | Histidine kinase, Bacterial chemotaxis sensory transducer | | | | | | | | COG0840 | | | | | |
| swp1499 | | | 0.396 | | | Flagellar basal-body rod protein FlgC | | | | | | | | COG1558 | | | | | |
| swp5086 | | | 2.087 | | | Flagellar transport protein FliP | | | | | | | | COG1338 | | | | | |
| swp5082 | | | 2.102 | | | Type III secretion FHIPEP | | | | | | | | COG1298 | | | | | |
| swp5105 | | | 2.105 | | | Flagellar basal-body rod protein FlgB | | | | | | | | COG1815 | | | | | |
| swp5106 | | | 2.148 | | | Flagellar basal-body rod protein FlgC | | | | | | | | COG1558 | | | | | |
| swp5120 | | | 2.171 | | | Flagellar protein FliS | | | | | | | | COG1516 | | | | | |
| swp5122 | | | 2.308 | | | Flagellar hook-length control protein | | | | | | | | COG3144 | | | | | |
| swp5111 | | | 2.345 | | | Flagellar L-ring protein | | | | | | | | COG2063 | | | | | |
| swp5114 | | | 2.422 | | | Flagellar hook-associated protein, putative | | | | | | | | COG1256 | | | | | |
| swp5094 | | | 2.826 | | | Flagellar assembly protein FliH | | | | | | | | COG1317 | | | | | |
| swp5109 | | | 2.957 | | | Flagellar basal body rod protein | | | | | | | | COG4786 | | | | | |
| swp2979 | | | 3.242 | | | Histidine kinase, Bacterial chemotaxis sensory transducer | | | | | | | | COG0840 | | | | | |
| swp5110 | | | 3.722 | | | Flagellar basal body rod protein | | | | | | | | COG4786 | | | | | |
| swp5108 | | | 3.798 | | | Flagellar basal body rod protein FlaE | | | | | | | | COG1749 | | | | | |
| swp5107 | | | 3.879 | | | Flagellar hook capping protein | | | | | | | | COG1843 | | | | | |
| swp5118 | | | 10.157 | | | Flagellin, C-terminal:Flagellin, N-terminal | | | | | | | | COG1344 | | | | | |
| Intracellular trafficking, secretion and vesicular transport [U] | | | | | | | | | | | | | | | 5/49 | | |  | |
| swp0447 | | | 0.301 | | | Twin-arginine translocation protein TatB | | | | | | | | COG1826 | | | | | |
| swp2040 | | | 0.379 | | | Heme exporter protein CcmD | | | | | | | | COG3114 | | | | | |
| swp3980 | | | 0.387 | | | MotA/TolQ/ExbB proton channel | | | | | | | | COG0811 | | | | | |
| swp0192 | | | 0.490 | | | General secretion pathway protein J | | | | | | | | COG4795 | | | | | |
| swp3205 | | | 4.037 | | | Biopolymer transport protein ExbD/TolR | | | | | | | | COG0848 | | | | | |
| Posttranslational modification, protein turnover, chaperones [O] | | | | | | | | | | | | | | | 23/162 | | |  | |
| swp2041 | | 0.218 | | | | | | Cytochrome c-type biogenesis protein CcmC | | | | | | | COG0755 | | |  | |
| swp4043 | | 0.248 | | | | | | DSBA oxidoreductase | | | | | | | COG0526 | | |  | |
| swp2844 | | 0.271 | | | | | | Heat shock protein Hsp20 | | | | | | | COG0071 | | |  | |
| swp3164 | | 0.271 | | | | | | Formate acetyltransferase activating enzyme | | | | | | | COG0602 | | |  | |
| swp2042 | | 0.320 | | | | | | Cytochrome c-type biogenesis protein CcmB | | | | | | | COG2386 | | |  | |
| swp1951 | | 0.349 | | | | | | Pyruvate formate-lyase 1 activating enzyme | | | | | | | COG1180 | | |  | |
| swp0977 | | 0.371 | | | | | | Protein-P-II uridylyltransferase | | | | | | | COG2844 | | |  | |
| swp4658 | | 0.375 | | | | | | Cytochrome c-type biogenesis protein CcmF | | | | | | | COG1138 | | |  | |
| swp1260 | | 0.381 | | | | | | Peptidase U32 | | | | | | | COG0826 | | |  | |
| swp2924 | | 0.381 | | | | | | Peptidase M48, Ste24p:HtpX, N-terminal | | | | | | | COG0501 | | |  | |
| swp1871 | | 0.408 | | | | | | ATP-dependent Clp protease, ATP-binding subunit ClpA | | | | | | | COG0542 | | |  | |
| swp5099 | | 0.447 | | | | | | Glutathione S-transferase | | | | | | | COG0625 | | |  | |
| swp1427 | | 0.461 | | | | | | Quinol oxidase subunit II | | | | | | | COG0109 | | |  | |
| swp2039 | | 0.464 | | | | | | CcmE/CycJ protein | | | | | | | COG2332 | | |  | |
| swp3018 | | 0.468 | | | | | | Conserved hypothetical protein | | | | | | | COG4067 | | |  | |
| swp0214 | | 0.476 | | | | | | Radical activating enzyme | | | | | | | COG1180 | | |  | |
| swp2647 | | 0.495 | | | | | | Hydrogenase formation HypD protein | | | | | | | COG0409 | | |  | |
| swp0254 | | 2.129 | | | | | | Glutathione S-transferase, N-terminal | | | | | | | COG0625 | | |  | |
| swp2807 | | 2.187 | | | | | | Peptidyl-prolyl cis-trans isomerase, cyclophilin type | | | | | | | COG0652 | | |  | |
| swp3262 | | 2.292 | | | | | | Trigger factor | | | | | | | COG0544 | | |  | |
| swp2574 | | 2.725 | | | | | | Cbb3-type cytochrome oxidase component | | | | | | | COG4736 | | |  | |
| swp4327 | | 7.050 | | | | | | Alkyl hydroperoxide reductase/ Thiol specific antioxidant/ Mal allergen | | | | | | | COG0450 | | |  | |
| swp4326 | | 8.869 | | | | | | FAD-dependent pyridine nucleotide-disulphide oxidoreductase | | | | | | | COG3634 | | |  | |
| **Metabolism** | | | | | | | | | | | | | | | **169/1139** | | |  | |
| Energy production and conversion [C] | | | | | | | | | | | | | | | 52/239 | | |  | |
| swp0430 | | | 0.096 | | | Fumarate reductase flavoprotein subunit | | | | | | | | COG1053 | | |  | | |
| swp0429 | | | 0.139 | | | Fumarate reductase respiratory complex, transmembrane subunit | | | | | | | | COG2009 | | |  | | |
| swp4014 | | | 0.164 | | | Respiratory NADH dehydrogenase II | | | | | | | | COG1252 | | |  | | |
| swp1739 | | | 0.222 | | | Sodium:dicarboxylate symporter | | | | | | | | COG1301 | | |  | | |
| swp3631 | | | 0.230 | | | L-lactate permease | | | | | | | | COG1620 | | |  | | |
| swp2640 | | | 0.261 | | | Nickel-dependent hydrogenase, large subunit | | | | | | | | COG0374 | | |  | | |
| swp4177 | | | 0.262 | | | Aldehyde dehydrogenase (NAD+) | | | | | | | | COG1012 | | |  | | |
| swp1422 | | | 0.266 | | | Cytochrome o ubiquinol oxidase subunit II | | | | | | | | COG1622 | | |  | | |
| swp2053 | | | 0.275 | | | Phosphoenolpyruvate carboxylase | | | | | | | | COG2352 | | |  | | |
| swp4046 | | | 0.288 | | | Protoporphyrinogen oxidase | | | | | | | | COG4635 | | |  | | |
| swp0428 | | | 0.325 | | | Fumarate reductase cytochrome B subunit | | | | | | | | COG2009 | | |  | | |
| swp1311 | | | 0.341 | | | Alcohol dehydrogenase II | | | | | | | | COG1454 | | |  | | |
| swp4555 | | | 0.345 | | | Cytochrome c, class I | | | | | | | | COG2863 | | |  | | |
| swp2889 | | | 0.389 | | | NADH:ubiquinone oxidoreductase, Na(+)-translocating, A subunit | | | | | | | | COG1726 | | |  | | |
| swp5036 | | | 0.390 | | | Formate dehydrogenase, subunit FdhD | | | | | | | | COG1526 | | |  | | |
| swp4116 | | | 0.394 | | | 4Fe-4S ferredoxin, iron-sulfur binding | | | | | | | | COG0437 | | |  | | |
| swp5029 | | | 0.398 | | | Twin-arginine translocation pathway signal | | | | | | | | COG0243 | | |  | | |
| swp4554 | | | 0.401 | | | Cytochrome c family protein | | | | | | | | COG3258 | | |  | | |
| swp1425 | | | 0.406 | | | Cytochrome c oxidase, subunit III | | | | | | | | COG1845 | | |  | | |
| swp0969 | | | 0.412 | | | Succinate-semialdehyde dehydrogenase | | | | | | | | COG1012 | | |  | | |
| swp4047 | | | 0.413 | | | Thiol oxidoreductase | | | | | | | | COG3488 | | |  | | |
| swp1892 | | | 0.422 | | | Blue (type 1) copper domain | | | | | | | | COG3241 | | |  | | |
| swp2629 | | | 0.450 | | | Isocitrate lyase and phosphorylmutase | | | | | | | | COG2224 | | |  | | |
| swp5025 | | | 0.451 | | | | | | | | | Twin-arginine translocation pathway signal | | | | COG0243 | | |  |
| swp4258 | | | 0.468 | | | | | | | | | Sodium:dicarboxylate symporter | | | | COG1301 | | |  |
| swp0051 | | | 0.491 | | | | | | | | | Protoporphyrinogen oxidase | | | | COG4635 | | |  |
| swp1470 | | | 2.031 | | | | | | | | | Cytochrome d ubiquinol oxidase, subunit II | | | | COG1294 | | |  |
| swp0725 | | | 2.101 | | | | | | | | | 4Fe-4S ferredoxin, iron-sulfur binding | | | | COG0437 | | |  |
| swp3875 | | | 2.155 | | | | | | | | | Malate dehydrogenase (oxaloacetate decarboxylating) (NADP^+^) | | | | COG0281 | | |  |
| swp0795 | | | 2.323 | | | | | | | | | Ubiquinol-cytochrome c reductase, cytochrome c1 | | | | COG2857 | | |  |
| swp4662 | | | 2.398 | | | | | | | | | Na^+^/H^+^ antiporter NhaC | | | | COG1757 | | |  |
| swp1949 | | | 2.416 | | | | | | | | | Acetate kinase | | | | COG0282 | | |  |
| swp5095 | | | 2.515 | | | | | | | | | ATPase FliI/YscN | | | | COG0055 | | |  |
| swp2573 | | | 2.539 | | | | | | | | | Cytochrome c oxidase, mono-heme subunit/FixO | | | | COG2993 | | |  |
| swp1170 | | | 2.681 | | | | | | | | | NADH:ubiquinone oxidoreductase, Na(+)-translocating, D subunit | | | | COG4660 | | |  |
| swp1695 | | | 2.700 | | | | | | | | | FeS cluster assembly scaffold IscU | | | | COG0822 | | |  |
| swp0256 | | | 2.749 | | | | | | | | | Iron-containing alcohol dehydrogenase | | | | COG1454 | | |  |
| swp3755 | | | 2.850 | | | | | | | | | Cytochrome c, class II | | | | COG3909 | | |  |
| swp2143 | | | 2.915 | | | | | | | | | Twin-arginine translocation pathway signal | | | | COG0243 | | |  |
| swp3606 | | | 2.953 | | | | | | | | | Isocitrate dehydrogenase NAD-dependent, mitochondrial | | | | COG0473 | | |  |
| swp1167 | | | 2.992 | | | | | | | | | NADH:ubiquinone oxidoreductase, Na(+)-translocating, A subunit | | | | COG1726 | | |  |
| swp5158 | | | 3.039 | | | | | | | | | ATP synthase F1, alpha subunit | | | | COG0056 | | |  |
| swp5162 | | | 3.117 | | | | | | | | | H^+^-transporting two-sector ATPase, A subunit | | | | COG0356 | | |  |
| swp5159 | | | 3.139 | | | | | | | | | H^+^-transporting two-sector ATPase, delta (OSCP) subunit | | | | COG0712 | | |  |
| swp5160 | | | 3.183 | | | | | | | | | ATP synthase F0, subunit B | | | | COG0711 | | |  |
| swp5157 | | | 3.302 | | | | | | | | | ATP synthase F1, gamma subunit | | | | COG0224 | | |  |
| swp0724 | | | 3.676 | | | | | | | | | Twin-arginine translocation pathway signal | | | | COG0243 | | |  |
| swp1169 | | | 4.708 | | | | | | | | | NADH:ubiquinone oxidoreductase, Na(+)-translocating, C subunit | | | | COG2869 | | |  |
| swp1813 | | | 7.002 | | | | | | | | | L-lactate permease | | | | COG1620 | | |  |
| swp3459 | | | 7.073 | | | | | | | | | Twin-arginine translocation pathway signal | | | | COG0243 | | |  |
| swp2139 | | | 7.885 | | | | | | | | | Formate dehydrogenase, gamma subunit | | | | COG2864 | | |  |
| swp2142 | | | 10.380 | | | | | | | | | 4Fe-4S ferredoxin, iron-sulfur binding | | | | COG0437 | | |  |
| swp3458 | | | 10.387 | | | | | | | | | Anaerobic dimethyl sulfoxide reductase, B subunit | | | | COG0437 | | |  |
| Carbohydrate transport and metabolism [G] | | | | | | | | | | | | | | | 10/122 | | |  | |
| swp3297 | 0.150 | | | | | | | | | | Phosphoglucomutase/phosphomannomutase family protein | | | | COG1109 | | |  | |
| swp4289 | 0.200 | | | | | | | | | | Glucosamine-6-phosphate isomerase | | | | COG0363 | | |  | |
| swp0201 | 0.256 | | | | | | | | | | Phospholipid/glycerol acyltransferase | | | | COG0477 | | |  | |
| swp3701 | 0.305 | | | | | | | | | | N-acetylglucosamine-6-phosphate deacetylase | | | | COG1820 | | |  | |
| swp4527 | 0.313 | | | | | | | | | | Galactokinase | | | | COG0153 | | |  | |
| swp4782 | 0.384 | | | | | | | | | | Drug resistance transporter, Bcr/CflA family protein | | | | COG0477 | | |  | |
| swp4528 | 0.445 | | | | | | | | | | Aldose 1-epimerase | | | | COG2017 | | |  | |
| swp4709 | 0.470 | | | | | | | | | | Drug resistance transporter, EmrB/QacA subfamily | | | | COG0477 | | |  | |
| swp1851 | 2.227 | | | | | | | | | | Phosphoenolpyruvate synthase | | | | COG0574 | | |  | |
| swp0526 | 3.360 | | | | | | | | | | Glyceraldehyde-3-phosphate dehydrogenase, type I | | | | COG0057 | | |  | |
| Amino acid transport and metabolism [E] | | | | | | | | | | | | | | | 22/250 | | |  | |
| swp4262 | 0.106 | | | | | | | | | | Iron-sulfur-dependent L-serine dehydratase single chain form | | | | COG1760 | | |  | |
| swp1163 | 0.193 | | | | | | | | | | Amino acid-binding ACT | | | | COG2716 | | |  | |
| swp4263 | 0.218 | | | | | | | | | | Serine transporter, putative | | | | COG0814 | | |  | |
| swp3588 | 0.313 | | | | | | | | | | Di- and tripeptidases | | | | COG2195 | | |  | |
| swp1428 | 0.322 | | | | | | | | | | Peptidyl-dipeptidase Dcp | | | | COG0339 | | |  | |
| swp4162 | 0.341 | | | | | | | | | | FAD dependent oxidoreductase | | | | COG0665 | | |  | |
| swp2043 | 0.423 | | | | | | | | | | Heme exporter protein CcmA | | | | COG1126 | | |  | |
| swp2698 | 0.429 | | | | | | | | | | Proteobacterial succinyl-diaminopimelate desuccinylase | | | | COG0624 | | |  | |
| swp2941 | 0.439 | | | | | | | | | | Glutamate synthase domain protein | | | | COG0069 | | |  | |
| swp3003 | 0.439 | | | | | | | | | | Histidinol dehydrogenase | | | | COG0141 | | |  | |
| swp2055 | 0.441 | | | | | | | | | | N-acetyl-gamma-glutamyl-phosphate reductase | | | | COG0002 | | |  | |
| swp2392 | 0.464 | | | | | | | | | | 6-phosphogluconate dehydratase | | | | COG0129 | | |  | |
| swp5051 | 0.492 | | | | | | | | | | Sodium/glutamate symporter | | | | COG0786 | | |  | |
| swp3004 | 0.498 | | | | | | | | | | Histidinol-phosphate aminotransferase | | | | COG0079 | | |  | |
| swp1919 | 2.011 | | | | | | | | | | Tryptophan synthase, beta chain | | | | COG0133 | | |  | |
| swp2632 | 2.044 | | | | | | | | | | Peptidase | | | | COG1506 | | |  | |
| swp1555 | 2.166 | | | | | | | | | | Amino acid/peptide transporter | | | | COG3104 | | |  | |
| swp4219 | 2.279 | | | | | | | | | | Leucyl aminopeptidase | | | | COG0260 | | |  | |
| swp0709 | 2.743 | | | | | | | | | | Prolyl oligopeptidase | | | | COG1505 | | |  | |
| swp1694 | 2.832 | | | | | | | | | | Cysteine desulfurase IscS | | | | COG1104 | | |  | |
| swp1202 | 3.121 | | | | | | | | | | Carbamoyl-phosphate synthase, large subunit | | | | COG0458 | | |  | |
| swp2282 | 5.898 | | | | | | | | | | Peptidyl-dipeptidase Dcp | | | | COG0339 | | |  | |
| Nucleotide transport and metabolism [F] | | | | | | | | | | | | | | | 13/65 | | |  | |
| swp3165 | 0.090 | | | | | | | | | | Ribonucleoside-triphosphate reductase | | | | COG1328 | | |  | |
| swp2520 | 0.176 | | | | | | | | | | Metallophosphoesterase:5'-Nucleotidase, C-terminal | | | | COG0737 | | |  | |
| swp2621 | 0.337 | | | | | | | | | | Deoxyguanosinetriphosphate triphosphohydrolase | | | | COG0232 | | |  | |
| swp4436 | 0.352 | | | | | | | | | | Nucleoside permease NupC | | | | COG1972 | | |  | |
| swp3037 | 0.357 | | | | | | | | | | Cytidine/deoxycytidylate deaminase, zinc-binding region | | | | COG0295 | | |  | |
| swp0551 | 0.431 | | | | | | | | | | Formate--tetrahydrofolate ligase | | | | COG2759 | | |  | |
| swp1230 | 0.480 | | | | | | | | | | Thymidine phosphorylase | | | | COG0213 | | |  | |
| swp3520 | 2.089 | | | | | | | | | | Uridylate kinase | | | | COG0528 | | |  | |
| swp0704 | 2.236 | | | | | | | | | | Adenylosuccinate synthetase | | | | COG0104 | | |  | |
| swp2478 | 2.355 | | | | | | | | | | Ribonucleoside-diphosphate reductase | | | | COG0209 | | |  | |
| swp3112 | 2.490 | | | | | | | | | | Amidophosphoribosyltransferase | | | | COG0034 | | |  | |
| swp1817 | 3.144 | | | | | | | | | | Phosphoribosylglycinamide formyltransferase | | | | COG0299 | | |  | |
| swp1463 | 5.434 | | | | | | | | | | IMP dehydrogenase | | | | COG0516 | | |  | |
| Coenzyme transport and metabolism [H] | | | | | | | | | | | | | | | 18/129 | | |  | |
| swp5008 | 0.134 | | | | | | | | | | | | Molybdenum cofactor biosynthesis protein | | COG0521 | | |  | |
| swp5079 | 0.148 | | | | | | | | | | | | Oxygen-independent coproporphyrinogen III oxidase | | COG0635 | | |  | |
| swp5006 | 0.188 | | | | | | | | | | | | Molybdenum cofactor biosynthesis protein D | | COG1977 | | |  | |
| swp5018 | 0.204 | | | | | | | | | | | | Oxygen-independent coproporphyrinogen III oxidase | | COG0635 | | |  | |
| swp3985 | 0.239 | | | | | | | | | | | | Coproporphyrinogen III oxidase, putative | | COG0635 | | |  | |
| swp5009 | 0.239 | | | | | | | | | | | | Radical SAM:Molybdenum cofactor synthesis C | | COG2896 | | |  | |
| swp5007 | 0.241 | | | | | | | | | | | | Molybdopterin cofactor biosynthesis protein MoaC | | COG0315 | | |  | |
| swp1736 | 0.242 | | | | | | | | | | | | Ferrochelatase | | COG0276 | | |  | |
| swp5005 | 0.262 | | | | | | | | | | | | Molybdenum cofactor biosynthesis protein E | | COG0314 | | |  | |
| swp4178 | 0.324 | | | | | | | | | | | | Aminotransferase, class III | | COG0161 | | |  | |
| swp0165 | 0.413 | | | | | | | | | | | | Molybdopterin biosynthesis MoeB protein | | COG0476 | | |  | |
| swp4476 | 0.425 | | | | | | | | Glutathione synthetase, prokaryotic | | | | | | COG0189 | | |  | |
| swp0166 | 0.427 | | | | | | | | Molybdenum cofactor biosynthesis protein | | | | | | COG0303 | | |  | |
| swp1390 | 0.478 | | | | | | | | Glutamate--cysteine ligase, monofunctional | | | | | | COG2918 | | |  | |
| swp3945 | 2.568 | | | | | | | | TonB-dependent vitamin B12 receptor | | | | | | COG4206 | | |  | |
| swp4642 | 3.021 | | | | | | | | Oxidoreductase FAD/NAD(P)-binding | | | | | | COG0543 | | |  | |
| swp0169 | 3.584 | | | | | | | | 3,4-Dihydroxy-2-butanone 4-phosphate synthase | | | | | | COG0108 | | |  | |
| swp4059 | 4.826 | | | | | | | | Uroporphyrin-III C-methyltransferase, C-terminal | | | | | | COG0007 | | |  | |
| Lipid transport and metabolism [I] | | | | | | | | | | | | | | | 15/120 | | |  | |
| swp2982 | 0.140 | | | | | | | | Acyl-CoA dehydrogenase, C-terminal | | | | | | COG1960 | | |  | |
| swp2312 | 0.280 | | | | | | | | Acyl-CoA dehydrogenase, C-terminal | | | | | | COG1960 | | |  | |
| swp0563 | 0.428 | | | | | | | | Enoyl-CoA hydratase/isomerase | | | | | | COG1024 | | |  | |
| swp3443 | 0.440 | | | | | | | | Propionyl-CoA carboxylase | | | | | | COG4799 | | |  | |
| swp3442 | 0.461 | | | | | | | | Isovaleryl-CoA dehydrogenase | | | | | | COG1960 | | |  | |
| swp3400 | 0.462 | | | | | | | | Short-chain dehydrogenase/reductase SDR | | | | | | COG1028 | | |  | |
| swp0204 | 0.466 | | | | | | | | Fatty acid desaturase, family 1 | | | | | | COG1398 | | |  | |
| swp0114 | 0.480 | | | | | | | | AMP-dependent synthetase and ligase | | | | | | COG0318 | | |  | |
| swp0315 | 2.115 | | | | | | | | AMP-dependent synthetase and ligase | | | | | | COG0318 | | |  | |
| swp4935 | 2.516 | | | | | | | | Probable crotonobetaine/carnitine-CoA ligase | | | | | | COG0318 | | |  | |
| swp3043 | 2.710 | | | | | | | | Acyl carrier protein (ACP) | | | | | | COG0236 | | |  | |
| swp2802 | 2.945 | | | | | | | | Beta-hydroxyacyl-(acyl-carrier-protein) dehydratase FabA | | | | | | COG0764 | | |  | |
| swp3153 | 3.331 | | | | | | | | Membrane protein involved in aromatic hydrocarbon degradation | | | | | | COG2067 | | |  | |
| swp2217 | 4.854 | | | | | | | | Long-chain fatty acid transport protein | | | | | | COG2067 | | |  | |
| swp3121 | 6.844 | | | | | | | | Beta-ketoacyl synthase | | | | | | COG0304 | | |  | |
| Inorganic ion transport and metabolism [P] | | | | | | | | | | | | | | | 37/172 | | |  | |
| swp3978 | 0.073 | | | | | | | | TonB-dependent haemoglobin/transferrin/lactoferrin receptor | | | | | | COG1629 | | |  | |
| swp3568 | 0.080 | | | | | | | | TonB-dependent receptor | | | | | | COG1629 | | |  | |
| swp0161 | 0.088 | | | | | | | | TonB-dependent receptor | | | | | | COG1629 | | |  | |
| swp2362 | 0.092 | | | | | | | | TonB-dependent receptor | | | | | | COG1629 | | |  | |
| swp3982 | 0.101 | | | | | | | | Periplasmic binding protein | | | | | | COG0614 | | |  | |
| swp3270 | 0.106 | | | | | | | | Ferrous iron transport protein B | | | | | | COG0370 | | |  | |
| swp1134 | 0.137 | | | | | | | | TonB-dependent receptor | | | | | | COG1629 | | |  | |
| swp3974 | 0.148 | | | | | | | | Pyridoxamine 5'-phosphate oxidase-related | | | | | | COG0748 | | |  | |
| swp3976 | 0.155 | | | | | | | | Conserved hypothetical protein | | | | | | COG3721 | | |  | |
| swp3984 | 0.158 | | | | | | | | ABC transporter | | | | | | COG1120 | | |  | |
| swp3983 | 0.168 | | | | | | | | Transport system permease protein | | | | | | COG0609 | | |  | |
| swp3303 | 0.187 | | | | | | | | TonB-dependent receptor | | | | | | COG1629 | | |  | |
| swp4048 | 0.222 | | | | | | | | Iron-regulated protein A precursor | | | | | | COG3487 | | |  | |
| swp3271 | 0.265 | | | | | | | | FeoA | | | | | | COG1918 | | |  | |
| swp5004 | 0.269 | | | | | | | | Molybdenum ABC transporter | | | | | | COG0725 | | |  | |
| swp0926 | 0.287 | | | | | | | | TonB-dependent siderophore receptor | | | | | | COG1629 | | |  | |
| swp0953 | 0.300 | | | | | | | | TonB-dependent haem/haemoglobin receptor | | | | | | COG1629 | | |  | |
| swp4980 | 0.301 | | | | | | | | Binding-protein-dependent transport systems inner membrane component | | | | | | COG4149 | | |  | |
| swp2100 | 0.323 | | | | | | | | Ferrichrome-binding protein | | | | | | COG0614 | | |  | |
| swp2097 | 0.350 | | | | | | | | Ferrichrome-iron receptor | | | | | | COG1629 | | |  | |
| swp4971 | 0.366 | | | | | | | | TonB-dependent receptor | | | | | | COG1629 | | |  | |
| swp0995 | 0.371 | | | | | | | | Transport system permease protein | | | | | | COG0609 | | |  | |
| swp5037 | 0.399 | | | | | | | | Excisionase/Xis, DNA-binding | | | | | | COG1910 | | |  | |
| swp4055 | 0.406 | | | | | | | | Adenylylsulfate kinase | | | | | | COG0529 | | |  | |
| swp2099 | 0.421 | | | | | | | | Ferrichrome transport ATP-binding protein FhuC | | | | | | COG1120 | | |  | |
| swp4139 | 0.466 | | | | | | | | Outer membrane protein | | | | | | COG1629 | | |  | |
| swp4477 | 0.496 | | | | | | | | Cold-active alkaline phosphatase | | | | | | COG1785 | | |  | |
| swp0853 | 2.177 | | | | | | | | Nitrite/sulfite reductase | | | | | | COG0155 | | |  | |
| swp0525 | 2.217 | | | | | | | | Bile acid:sodium symporter | | | | | | COG0798 | | |  | |
| swp1591 | 2.248 | | | | | | | | Transporter, putative | | | | | | COG0471 | | |  | |
| swp2701 | 2.726 | | | | | | | | Carbonic anhydrase, prokaryotic and plant | | | | | | COG0288 | | |  | |
| swp1592 | 2.908 | | | | | | | | Adenylylsulfate kinase | | | | | | COG0529 | | |  | |
| swp2866 | 2.955 | | | | | | | | Cytochrome-c peroxidase | | | | | | COG1858 | | |  | |
| swp3842 | 3.023 | | | | | | | | Outer membrane receptor protein | | | | | | COG1629 | | |  | |
| swp0851 | 4.134 | | | | | | | | Sulphite reductase (NADPH) flavoprotein, alpha chain | | | | | | COG0369 | | |  | |
| swp4022 | 9.653 | | | | | | | | TonB-dependent receptor | | | | | | COG1629 | | |  | |
| swp3548 | 11.484 | | | | | | | | TonB-dependent receptor | | | | | | COG1629 | | |  | |
| Secondary metabolites biosynthesis, transport and catabolism [Q] | | | | | | | | | | | | | | | 2/42 | | |  | |
| swp2926 | 0.452 | | | | | | | | Biotin synthesis protein BioC | | | | | | COG0500 | | |  | |
| swp3475 | 2.910 | | | | | | | | Amidohydrolase | | | | | | COG1228 | | |  | |
| **Poorly Characterized** | | | | | | | | | | | | | | | **56/575** | | |  | |
| General function prediction only [R] | | | | | | | | | | | | | | | 26/298 | | |  | |
| swp0958 | 0.050 | | | | | | | | CBS domain protein | | | | | | COG0517 | | |  | |
| swp0615 | 0.307 | | | | | | | | Conserved hypothetical protein | | | | | | COG1342 | | |  | |
| swp4956 | 0.330 | | | | | | | | Probable zinc protease | | | | | | COG0612 | | |  | |
| swp3143 | 0.339 | | | | | | | | Conserved hypothetical protein | | | | | | COG1721 | | |  | |
| swp3802 | 0.356 | | | | | | | | Fe-S oxidoreductase, putative | | | | | | COG1242 | | |  | |
| swp0891 | 0.406 | | | | | | | | Azoreductase, putative | | | | | | COG0431 | | |  | |
| swp0756 | 0.422 | | | | | | | | Sel1-like repeat | | | | | | COG0790 | | |  | |
| swp2765 | 0.439 | | | | | | | | Conserved hypothetical protein | | | | | | COG1721 | | |  | |
| swp4218 | 0.445 | | | | | | | | Permease YjgP/YjgQ | | | | | | COG0795 | | |  | |
| swp3145 | 0.456 | | | | | | | | Von Willebrand factor type A domain protein | | | | | | COG2304 | | |  | |
| swp3667 | 0.479 | | | | | | | | Rossmann fold nucleotide-binding protein, putative | | | | | | COG1611 | | |  | |
| swp0831 | 0.490 | | | | | | | | Insulinase-like:Peptidase M16, C-terminal | | | | | | COG0612 | | |  | |
| swp3554 | 0.498 | | | | | | | | Polyunsaturated fatty acid synthase PfaD | | | | | | COG2070 | | |  | |
| swp1400 | 2.026 | | | | | | | | Peptidase S41 | | | | | | COG2319 | | |  | |
| swp4026 | 2.199 | | | | | | | | Membrane protein, putative | | | | | | COG0701 | | |  | |
| swp1885 | 2.251 | | | | | | | | Rhodanese-like | | | | | | COG1054 | | |  | |
| swp0797 | 2.252 | | | | | | | | Stringent starvation protein b | | | | | | COG2969 | | |  | |
| swp1810 | 2.270 | | | | | | | | Peptidase M28 | | | | | | COG2234 | | |  | |
| swp2813 | 2.419 | | | | | | | | Glycosyl hydrolase, BNR repeat | | | | | | COG4447 | | |  | |
| swp1063 | 2.645 | | | | | | | | Beta-lactamase-like | | | | | | COG2220 | | |  | |
| swp2398 | 2.816 | | | | | | | | Membrane protein, putative | | | | | | COG2194 | | |  | |
| swp0947 | 3.416 | | | | | | | | Peptidase S45, penicillin amidase | | | | | | COG2366 | | |  | |
| swp3457 | 4.795 | | | | | | | | Cytoplasmic chaperone TorD | | | | | | COG3381 | | |  | |
| swp2812 | 5.131 | | | | | | | | Exporter of the RND superfamily | | | | | | COG1033 | | |  | |
| swp1738 | 7.358 | | | | | | | | Hexapeptide-repeat containing-acetyltransferase | | | | | | COG0110 | | |  | |
| swp2141 | 7.632 | | | | | | | | Cytoplasmic chaperone TorD | | | | | | COG3381 | | |  | |
| Function unknown [S] | | | | | | | | | | | | | | | 3077 | | |  | |
| swp0897 | 0.186 | | | | | | | | Membrane protein, putative | | | | | | COG1584 | | |  | |
| swp2687 | 0.197 | | | | | | | | Transcription factor jumonji, jmjC | | | | | | COG2850 | | |  | |
| swp4136 | 0.203 | | | | | | | | PepSY-associated TM helix | | | | | | COG3182 | | |  | |
| swp1058 | 0.282 | | | | | | | | Conserved hypothetical protein | | | | | | COG3803 | | |  | |
| swp4013 | 0.298 | | | | | | | | Conserved hypothetical protein | | | | | | COG0397 | | |  | |
| swp3744 | 0.302 | | | | | | | | Hemerythrin | | | | | | COG3945 | | |  | |
| swp0326 | 0.342 | | | | | | | | Conserved hypothetical protein | | | | | | COG2135 | | |  | |
| swp2793 | 0.362 | | | | | | | | Conserved hypothetical protein | | | | | | COG3108 | | |  | |
| swp3414 | 0.380 | | | | | | | | PepSY-associated TM helix | | | | | | COG3182 | | |  | |
| swp2902 | 0.407 | | | | | | | | DoxX family protein | | | | | | COG2259 | | |  | |
| swp2151 | 0.440 | | | | | | | | Conserved hypothetical protein | | | | | | COG2930 | | |  | |
| swp4703 | 0.458 | | | | | | | | Conserved hypothetical protein | | | | | | COG2841 | | |  | |
| swp0159 | 0.483 | | | | | | | | PepSY-associated TM helix | | | | | | COG3182 | | |  | |
| swp0987 | 0.493 | | | | | | | | Membrane protein, putative | | | | | | COG4125 | | |  | |
| swp1458 | 2.021 | | | | | | | | Lipoprotein, putative | | | | | | COG4380 | | |  | |
| swp1453 | 2.034 | | | | | | | | Puttive membrane-associated protein with TPR-like domain | | | | | | COG2976 | | |  | |
| swp3680 | 2.072 | | | | | | | | Conserved hypothetical protein | | | | | | COG4805 | | |  | |
| swp1113 | 2.073 | | | | | | | | GatB/Yqey | | | | | | COG1610 | | |  | |
| swp1697 | 2.100 | | | | | | | | HesB/YadR/YfhF family protein | | | | | | COG0316 | | |  | |
| swp2326 | 2.179 | | | | | | | | DTW domain-containing protein | | | | | | COG3148 | | |  | |
| swp4841 | 2.269 | | | | | | | | HesB/YadR/YfhF:Nitrogen-fixing NifU, C-terminal | | | | | | COG0316 | | |  | |
| swp3637 | 2.382 | | | | | | | | Conserved hypothetical protein | | | | | | COG1556 | | |  | |
| swp0416 | 2.385 | | | | | | | | LemA protein | | | | | | COG1704 | | |  | |
| swp5080 | 2.386 | | | | | | | | Conserved hypothetical protein | | | | | | COG4805 | | |  | |
| swp3026 | 2.468 | | | | | | | | RNA binding S1 | | | | | | COG2996 | | |  | |
| swp3256 | 2.618 | | | | | | | | Short-chain alcohol dehydrogenase | | | | | | COG3007 | | |  | |
| swp1459 | 2.759 | | | | | | | | Lipoprotein, putative | | | | | | COG4259 | | |  | |
| swp0175 | 3.535 | | | | | | | | Lipoprotein, putative | | | | | | COG3595 | | |  | |
| swp4974 | 4.252 | | | | | | | | Conserved hypothetical protein | | | | | | COG1738 | | |  | |
| swp2821 | 5.310 | | | | | | | | YebG family protein | | | | | | COG3141 | | |  | |
| **no COG identified (Hypothetical protein)** | | | | | | | | | | | | | | | **117/1824** | | |  | |
| swp4513 | 0.048 | | | | | | | | | Porin, Gram-negative type | | | | | - | | |  | |
| swp0729 | 0.085 | | | | | | | | | Hypothetical protein | | | | | - | | |  | |
| swp3977 | 0.106 | | | | | | | | | Hypothetical protein | | | | | - | | |  | |
| swp4323 | 0.122 | | | | | | | | | Conserved hypothetical protein | | | | | - | | |  | |
| swp0722 | 0.127 | | | | | | | | | Transcriptional regulator, LysR family | | | | | - | | |  | |
| swp0542 | 0.134 | | | | | | | | | Conserved hypothetical protein | | | | | - | | |  | |
| swp0691 | 0.150 | | | | | | | | | Sensor histidine kinase | | | | | - | | |  | |
| swp0215 | 0.153 | | | | | | | | | Conserved hypothetical protein | | | | | - | | |  | |
| swp1054 | 0.153 | | | | | | | | | Conserved hypothetical protein | | | | | - | | |  | |
| swp3857 | 0.184 | | | | | | | | | Bax domain protein | | | | | - | | |  | |
| swp3360 | 0.195 | | | | | | | | | Conserved hypothetical protein | | | | | - | | |  | |
| swp2623 | 0.204 | | | | | | | | | | Iron-containing alcohol dehydrogenase | | | | - | | |  | |
| swp0532 | 0.214 | | | | | | | | | | Conserved hypothetical protein | | | | - | | |  | |
| swp0168 | 0.217 | | | | | | | | | | Sensory box protein | | | | - | | |  | |
| swp4784 | 0.233 | | | | | | | | | | Conserved hypothetical protein | | | | - | | |  | |
| swp4507 | 0.254 | | | | | | | | | | Endonuclease | | | | - | | |  | |
| swp3136 | 0.258 | | | | | | | | | | Conserved hypothetical protein | | | | - | | |  | |
| swp0783 | 0.260 | | | | | | | | | | YjeF protein | | | | - | | |  | |
| swp1953 | 0.262 | | | | | | | | | | Conserved hypothetical integral membrane protein | | | | - | | |  | |
| swp4368 | 0.265 | | | | | | | | | | Conserved hypothetical protein | | | | - | | |  | |
| swp4120 | 0.270 | | | | | | | | | | Regulatory protein, LysR | | | | - | | |  | |
| swp0782 | 0.275 | | | | | | | | | | Conserved hypothetical protein | | | | - | | |  | |
| swp5034 | 0.278 | | | | | | | | | | Conserved hypothetical protein | | | | - | | |  | |
| swp4508 | 0.291 | | | | | | | | | | Conserved hypothetical protein | | | | - | | |  | |
| swp3139 | 0.296 | | | | | | | | | | Enoyl-CoA hydratase/isomerase:3-hydroxyacyl-CoA dehydrogenase, | | | | - | | |  | |
| swp3040 | 0.298 | | | | | | | | | | Conserved hypothetical protein | | | | - | | |  | |
| swp4415 | 0.301 | | | | | | | | | | Cytosolic protein, putative | | | | - | | |  | |
| swp3550 | 0.304 | | | | | | | | | | Omega-3 polyunsaturated fatty acid synthase PfaA | | | | - | | |  | |
| swp4976 | 0.308 | | | | | | | | | | Hypothetical protein | | | | - | | |  | |
| swp5033 | 0.308 | | | | | | | | | | Conserved hypothetical protein | | | | - | | |  | |
| swp3268 | 0.333 | | | | | | | | | | Conserved hypothetical protein | | | | - | | |  | |
| swp0328 | 0.341 | | | | | | | | | | Conserved hypothetical protein | | | | - | | |  | |
| swp2867 | 0.341 | | | | | | | | | | Conserved hypothetical protein | | | | - | | |  | |
| swp1894 | 0.348 | | | | | | | | | | Conserved hypothetical protein | | | | - | | |  | |
| swp0327 | 0.349 | | | | | | | | | | Hypothetical protein | | | | - | | |  | |
| swp3166 | 0.350 | | | | | | | | | | Conserved hypothetical protein | | | | - | | |  | |
| swp3551 | 0.352 | | | | | | | | | | Omega-3 polyunsaturated fatty acid synthase PfaB | | | | - | | |  | |
| swp1671 | 0.358 | | | | | | | | | | Conserved hypothetical protein | | | | - | | |  | |
| swp4999 | 0.362 | | | | | | | | | | Conserved hypothetical protein | | | | - | | |  | |
| swp3362 | 0.375 | | | | | | | | | | Conserved hypothetical protein | | | | - | | |  | |
| swp3235 | 0.379 | | | | | | | | | | Conserved hypothetical protein | | | | - | | |  | |
| swp1406 | 0.380 | | | | | | | | | | Lipoprotein, putative | | | | - | | |  | |
| swp3819 | 0.380 | | | | | | | | | | Nickel-containing superoxide dismutase, putative | | | | - | | |  | |
| swp4983 | 0.382 | | | | | | | | | | Molybdenum cofactor biosynthesis protein | | | | - | | |  | |
| swp4511 | 0.389 | | | | | | | | | | Conserved hypothetical protein | | | | - | | |  | |
| swp5030 | 0.396 | | | | | | | | | | Twin-arginine translocation pathway signal | | | | - | | |  | |
| swp1085 | 0.398 | | | | | | | | | | Conserved hypothetical protein | | | | - | | |  | |
| swp0617 | 0.399 | | | | | | | | | | Conserved hypothetical protein | | | | - | | |  | |
| swp1244 | 0.400 | | | | | | | | | | Conserved hypothetical protein | | | | - | | |  | |
| swp4312 | 0.404 | | | | | | | | | | Formate dehydrogenase, alpha subunit | | | | - | | |  | |
| swp3820 | 0.405 | | | | | | | | | | Conserved hypothetical protein | | | | - | | |  | |
| swp3867 | 0.406 | | | | | | | | | | Conserved hypothetical protein | | | | - | | |  | |
| swp3791 | 0.415 | | | | | | | | | | Conserved hypothetical protein | | | | - | | |  | |
| swp2256 | 0.416 | | | | | | | | | | Conserved hypothetical protein | | | | - | | |  | |
| swp1506 | 0.417 | | | | | | | | | | Flagellar protein FlgJ | | | | - | | |  | |
| swp3361 | 0.421 | | | | | | | | | | Conserved hypothetical protein | | | | - | | |  | |
| swp2472 | 0.431 | | | | | | | | | | 2,4-dienoyl-CoA reductase, putative | | | | - | | |  | |
| swp4080 | 0.435 | | | | | | | | | | Conserved hypothetical protein | | | | - | | |  | |
| swp5017 | 0.436 | | | | | | | | | | Hypothetical protein | | | | - | | |  | |
| swp5026 | 0.436 | | | | | | | | | | Twin-arginine translocation pathway signal | | | | - | | |  | |
| swp0668 | 0.437 | | | | | | | | | | Conserved hypothetical protein | | | | - | | |  | |
| swp1231 | 0.441 | | | | | | | | | | Phosphopentomutase | | | | - | | |  | |
| swp4009 | 0.442 | | | | | | | | | | Conserved hypothetical protein | | | | - | | |  | |
| swp0455 | 0.444 | | | | | | | | | | Conserved hypothetical protein | | | | - | | |  | |
| swp3359 | 0.449 | | | | | | | | | | Hypothetical protein | | | | - | | |  | |
| swp4811 | 0.455 | | | | | | | | | | Secretion protein HlyD | | | | - | | |  | |
| swp2124 | 0.456 | | | | | | | | | | Transcriptional regulatory protein, C-terminal | | | | - | | |  | |
| swp3415 | 0.466 | | | | | | | | | | Conserved hypothetical protein | | | | - | | |  | |
| swp1663 | 0.473 | | | | | | | | | | Ada regulatory protein, putative | | | | - | | |  | |
| swp0894 | 0.477 | | | | | | | | | | Regulatory protein, LysR | | | | - | | |  | |
| swp1125 | 0.477 | | | | | | | | | | ATPase | | | | - | | |  | |
| swp4639 | 0.484 | | | | | | | | | | Conserved hypothetical protein | | | | - | | |  | |
| swp4049 | 0.486 | | | | | | | | | | Conserved hypothetical signal peptide protein | | | | - | | |  | |
| swp4309 | 0.495 | | | | | | | | | | Hypothetical protein | | | | - | | |  | |
| swp4367 | 0.495 | | | | | | | | | | Conserved hypothetical signal peptide protein | | | | - | | |  | |
| swp3023 | 2.056 | | | | | | | | | | Adenosylmethionine decarboxylase | | | | - | | |  | |
| swp4775 | 2.090 | | | | | | | | | | Conserved hypothetical protein | | | | - | | |  | |
| swp1937 | 2.104 | | | | | | | | | | Conserved hypothetical protein | | | | - | | |  | |
| swp0716 | 2.106 | | | | | | | | | | TonB-dependent receptor:Cna B-type | | | | - | | |  | |
| swp4254 | 2.112 | | | | | | | | | | GGDEF domain protein | | | | - | | |  | |
| swp2981 | 2.243 | | | | | | | | | | Hypothetical protein | | | | - | | |  | |
| swp1593 | 2.336 | | | | | | | | | | Acetyltransferase | | | | - | | |  | |
| swp3014 | 2.356 | | | | | | | | | | Phenylalanyl-tRNA synthetase, beta subunit | | | | - | | |  | |
| swp5096 | 2.372 | | | | | | | | | | Conserved hypothetical protein | | | | - | | |  | |
| swp4560 | 2.474 | | | | | | | | | | Outer membrane or exported, putative | | | | - | | |  | |
| swp1931 | 2.494 | | | | | | | | | | Conserved hypothetical protein | | | | - | | |  | |
| swp3159 | 2.525 | | | | | | | | | | Peptidase S8 and S53, | | | | - | | |  | |
| swp0218 | 2.621 | | | | | | | | | | Conserved hypothetical protein | | | | - | | |  | |
| swp3474 | 2.684 | | | | | | | | | | Amidohydrolase | | | | - | | |  | |
| swp0632 | 2.708 | | | | | | | | | | Conserved hypothetical protein | | | | - | | |  | |
| swp2383 | 2.718 | | | | | | | | | | Peptidyl-dipeptidase A | | | | - | | |  | |
| swp0634 | 2.730 | | | | | | | | | | PDZ/DHR/GLGF:Peptidase S41 | | | | - | | |  | |
| swp0395 | 2.749 | | | | | | | | | | Outer membrane adhesin like protein | | | | - | | |  | |
| swp4966 | 2.753 | | | | | | | | | | Conserved hypothetical protein | | | | - | | |  | |
| swp4758 | 2.806 | | | | | | | | | | Hypothetical protein | | | | - | | |  | |
| swp2135 | 2.966 | | | | | | | | | | Hypothetical protein | | | | - | | |  | |
| swp4965 | 2.985 | | | | | | | | | | Conserved hypothetical protein | | | | - | | |  | |
| swp3986 | 3.112 | | | | | | | | | | Conserved hypothetical protein | | | | - | | |  | |
| swp4091 | 3.165 | | | | | | | | | | Conserved hypothetical protein | | | | - | | |  | |
| swp2089 | 3.185 | | | | | | | | | | Conserved hypothetical protein | | | | - | | |  | |
| swp1574 | 3.188 | | | | | | | | | | Chain length regulator (capsular polysaccharide biosynthesis) | | | | - | | |  | |
| swp1597 | 3.213 | | | | | | | | | | Excinuclease ATPase subunit | | | | - | | |  | |
| swp2814 | 3.239 | | | | | | | | | | Conserved hypothetical protein | | | | - | | |  | |
| swp3280 | 3.370 | | | | | | | | | | Outer membrane protein precursor MtrB | | | | - | | |  | |
| swp1740 | 3.450 | | | | | | | | | | Ig-like, group 1 | | | | - | | |  | |
| swp4186 | 3.598 | | | | | | | | | | Conserved hypothetical protein | | | | - | | |  | |
| swp1020 | 3.754 | | | | | | | | | | Hypothetical protein | | | | - | | |  | |
| swp4341 | 3.876 | | | | | | | | | | Protease-associated PA | | | | - | | |  | |
| swp2138 | 4.809 | | | | | | | | | | Porin, Gram-negative type | | | | - | | |  | |
| swp0721 | 5.672 | | | | | | | | | | Decaheme cytochrome c | | | | - | | |  | |
| swp1948 | 7.193 | | | | | | | | | | Phosphate acetyltransferase | | | | - | | |  | |
| swp1812 | 7.439 | | | | | | | | | | Iron-sulfur cluster-binding protein | | | | - | | |  | |
| swp1964 | 8.017 | | | | | | | | | | Conserved hypothetical protein | | | | - | | |  | |
| swp4362 | 11.993 | | | | | | | | | | Conserved hypothetical protein | | | | - | | |  | |
| swp0896 | 12.239 | | | | | | | | | | Decaheme cytochrome c | | | | - | | |  | |
| swp2323 | 21.481 | | | | | | | | | | Conserved hypothetical protein | | | | - | | |  | |
| swp0384 | 23.430 | | | | | | | | | | Conserved hypothetical protein | | | | - | | |  | |
