# Supplementary material for: The regulatory function of LexA is temperature-dependent in the deep-sea bacterium Shewanella piezotolerans WP3
Source: Front Microbiol. 2015 Jun 18;6:627. doi: 10.3389/fmicb.2015.00627 (PMC4471891; doi:10.3389/fmicb.2015.00627)
Supplement: Supplementary file 7 [file Table_2.DOC]

**Table S2. Differentially expressed genes in WP3Δ*lexA* at 4°C.**

| **Functional category and gene locus designation** | | **Avg fold change (WP3Δ*lexA* /WP3)** | | | **Product and description** | | | **COG designation or**  **no. of relevant genes/**  **total no. of genes** | |  |
| --- | --- | --- | --- | --- | --- | --- | --- | --- | --- | --- |
| **Information storage and processing** | | | | | | | | **15/580** | |  |
| Translation, ribosomal structure and biogenesis[J] | | | | | | | | 3/177 | |  |
| swp3757 | | 0.248 | | | Ribosomal subunit interface protein | | | COG1544 | |  |
| swp3408 | | 0.393 | | | Ribosomal subunit interface protein | | | COG1544 | |  |
| swp0787 | | 0.429 | | | tRNA delta(2)-isopentenylpyrophosphate transferase | | | COG0324 | |  |
| Transcription[K] | | | | | | | | 5/219 | |  |
| swp3734 | | 0.274 | | | Regulatory protein, ArsR | | | COG0640 | |  |
| swp0380 | | 0.351 | | | Transcriptional regulator, TetR family | | | COG1309 | |  |
| swp3786 | | 0.408 | | | RNA polymerse sigmaE factor | | | COG1595 | |  |
| swp3212 | | 2.050 | | | Helicase c2 | | | COG1199 | |  |
| swp3249 | | 0.478 | | | Phage shock protein A | | | COG1842 | |  |
| Replication, recombination and repair [L] | | | | | | | | 6/182 | |  |
| swp3111 | | 2.134 | | | DNA topoisomerase III | | | COG0550 | |  |
| swp1346 | | 2.434 | | | DNA repair protein RecN | | | COG0497 | |  |
| swp1179 | | 2.964 | | | DNA-directed DNA polymerase | | | COG0389 | |  |
| swp2325 | | 4.181 | | | DNA polymerase III alpha subunit | | | COG0587 | |  |
| swp1366 | | 5.026 | | | RecA bacterial DNA recombination protein | | | COG0468 | |  |
| swp2324 | | 5.731 | | | Nucleotidyltransferase/DNA polymerase involved in DNA repair | | | COG0389 | |  |
| **Cellular processes and signaling** | | | | | | | | **20/827** | |  |
| Defense mechanisms [V] | | | | | | | | 2/79 | |  |
| swp0378 | | 0.323 | | | Acriflavin resistance protein | | | COG0841 | |  |
| swp2309 | | 2.620 | | | AcrB/AcrD/AcrF family protein | | | COG0841 | |  |
| Signal transduction mechanisms [T] | | | | | | | | 2/159 | |  |
| swp3785 | | 0.499 | | | Sigma-E factor negative regulatory protein | | | COG3073 | |  |
| swp2820 | | 2.289 | | | Putative GAF sensor protein | | | COG1956 | |  |
| Cell wall/membrane/envelope biogenesis [M] | | | | | | | | 7/214 | |  |
| swp4514 | | 0.211 | | | Porin, Gram-negative type | | | COG3203 | |  |
| swp0247 | | 0.303 | | | Outer membrane protein, putative | | | COG3637 | |  |
| swp3209 | | 0.324 | | | Outer membrane porin, putative | | | COG3203 | |  |
| swp3093 | | 0.377 | | | OmpA/MotB domain-contaning protein | | | COG2885 | |  |
| swp0248 | | 0.424 | | | OmpA-like transmembrane region | | | COG3637 | |  |
| swp3091 | | 0.439 | | | Peptidase C60, sortase A and B | | | COG3764 | |  |
| swp0379 | | 0.447 | | | HlyD family secretion protein | | | COG0845 | |  |
| Posttranslational modification, protein turnover, chaperones [O] | | | | | | | | 9/162 | |  |
| swp2844 | | 0.137 | | | Heat shock protein Hsp20 | | | COG0071 | |  |
| swp1732 | | 0.241 | | | Heat shock protein Hsp90:ATP-binding region, ATPase-like | | | COG0326 | |  |
| swp0464 | | 0.376 | | | 20S proteasome, A and B subunits | | | COG5405 | |  |
| swp0463 | | 0.384 | | | Heat shock protein HslU | | | COG1219 | |  |
| swp3018 | | 0.385 | | | Conserved hypothetical protein | | | COG4067 | |  |
| swp1196 | | 0.475 | | | Heat shock protein Hsp70 | | | COG0443 | |  |
| swp1734 | | 0.482 | | | Thioredoxin-like protein, putative | | | COG3118 | |  |
| swp2924 | | 0.490 | | | Peptidase M48, Ste24p:HtpX, N-terminal | | | COG0501 | |  |
| swp4043 | | 0.410 | | | DSBA oxidoreductase | | | COG0526 | |  |
| **Metabolism** | | | | | | | | **40/1139** | |  |
| Energy production and conversion [C] | | | | | | | | 13/239 | |  |
| swp1952 | | | | 0.168 | | | Formate acetyltransferase | | COG1882 | |
| swp1949 | | | | 0.246 | | | Acetate kinase | | COG0282 | |
| swp1469 | | | | 0.298 | | | Cytochrome bd ubiquinol oxidase, subunit I | | COG1271 | |
| swp0430 | | | | 0.321 | | | Fumarate reductase flavoprotein subunit | | COG1053 | |
| swp5023 | | | | 0.340 | | | Formate dehydrogenase, gamma subunit | | COG2864 | |
| swp0431 | | | | 0.357 | | | Succinate dehydrogenase/fumarate reductase iron-sulfur protein | | COG0479 | |
| swp3810 | | | | 0.393 | | | Betaine aldehyde dehydrogenase | | COG1012 | |
| swp1311 | | | | 0.420 | | | Alcohol dehydrogenase II | | COG1454 | |
| swp5024 | | | | 0.437 | | | Formate dehydrogenase, iron-sulfur subunit | | COG0437 | |
| swp5027 | | | | 0.444 | | | Formate dehydrogenase, gamma subunit | | COG2864 | |
| swp2053 | | | | 0.481 | | | Phosphoenolpyruvate carboxylase | | COG2352 | |
| swp5029 | | | | 0.498 | | | Twin-arginine translocation pathway signal | | COG0243 | |
| swp3635 | | | | 2.146 | | | Conserved hypothetical protein | | COG0247 | |
| Amino acid transport and metabolism [E] | | | | | | | | 2/250 | |  |
| swp4926 | | | 0.372 | | | Oligopeptidase A | | COG0339 | |  |
| swp2392 | | | 0.487 | | | 6-phosphogluconate dehydratase | | COG0129 | |  |
| Nucleotide transport and metabolism [F] | | | | | | | | 2/65 | |  |
| swp4077 | 0.453 | | | | | Phosphoribosylglycinamide formyltransferase 2 | | COG0026 | |  |
| swp3808 | 0.485 | | | | | Inosine/uridine-preferring nucleoside hydrolase | | COG1957 | |  |
| Coenzyme transport and metabolism [H] | | | | | | | | 3/129 | |  |
| swp2927 | | | 2.089 | | | 8-amino-7-oxononanoate synthase | | COG0156 | |  |
| swp2929 | | | 2.235 | | | Adenosylmethionine--8-amino-7-oxononanoate aminotransferase | | COG0161 | |  |
| swp2928 | | | 2.818 | | | Biotin synthase | | COG0502 | |  |
| Lipid transport and metabolism [I] | | | | | | | | 14/120 | |  |
| swp3140 | | | 0.248 | | | Thiolase | | COG0183 | |  |
| swp0034 | | | 0.305 | | | Thiolase | | COG0183 | |  |
| swp2312 | | | 0.312 | | | Acyl-CoA dehydrogenase, C-terminal | | COG1960 | |  |
| swp2982 | | | 0.331 | | | Acyl-CoA dehydrogenase, C-terminal | | COG1960 | |  |
| swp2386 | | | 0.439 | | | Acyl-CoA dehydrogenase, C-terminal | | COG1960 | |  |
| swp3385 | | | 2.062 | | | Butyryl-CoA dehydrogenase | | COG1960 | |  |
| swp3445 | | | 2.072 | | | Acetyl/propionyl-CoA carboxylase, alpha subunit | | COG4770 | |  |
| swp1842 | | | 2.309 | | | AMP-dependent synthetase and ligase | | COG0365 | |  |
| swp3383 | | | 2.464 | | | Enoyl-CoA hydratase/isomerase family protein | | COG1024 | |  |
| swp3384 | | | 2.934 | | | Enoyl-CoA hydratase/isomerase | | COG1024 | |  |
| swp3382 | | | 3.096 | | | 3-hydroxyisobutyrate dehydrogenase | | COG2084 | |  |
| swp3153 | | | 3.889 | | | Membrane protein involved in aromatic hydrocarbon degradation | | COG2067 | |  |
| swp2802 | | | 5.097 | | | Beta-hydroxyacyl-(acyl-carrier-protein) dehydratase FabA | | COG0764 | |  |
| swp3121 | | | 3.852 | | | Beta-ketoacyl synthase | | COG0304 | |  |
| Inorganic ion transport and metabolism [P] | | | | | | | | 5/172 | |  |
| swp4971 | | | 0.300 | | | TonB-dependent receptor | | COG1629 | |  |
| swp1175 | | | 0.378 | | | Bacterioferritin | | COG2193 | |  |
| swp3377 | | | 0.393 | | | Copper-translocating P-type ATPase | | COG2217 | |  |
| swp4592 | | | 2.143 | | | TonB-dependent receptor | | COG1629 | |  |
| swp4022 | | | 2.241 | | | TonB-dependent receptor | | COG1629 | |  |
| Secondary metabolites biosynthesis, transport and catabolism [Q] | | | | | | | | 1/42 | |  |
| swp2926 | | 2.214 | | | Biotin synthesis protein BioC | | | COG0500 | |  |
| **Poorly Characterized** | | | | | | | | **6/575** | |  |
| General function prediction only [R] | | | | | | | | 2/298 | |  |
| swp0958 | | 0.141 | | | CBS domain protein | | | COG0517 | |  |
| swp4181 | | 0.447 | | | Enzyme of the cupin superfamily, putative | | | COG3450 | |  |
| Function unknown [S] | | | | | | | | 4/277 | |  |
| swp4485 | | 0.316 | | | Conserved hypothetical protein | | | COG3422 | |  |
| swp1471 | | 0.316 | | | Conserved hypothetical protein | | | COG4890 | |  |
| swp3256 | | 2.085 | | | Short-chain alcohol dehydrogenase | | | COG3007 | |  |
| swp2821 | | 3.287 | | | YebG family protein | | | COG3141 | |  |
| **no COG identified (Hypothetical protein)** | | | | | | | | **28/1824** | |  |
| swp3092 | | 0.184 | | | Von Willebrand factor, type A | | | - | |  |
| swp3040 | | 0.189 | | | Conserved hypothetical protein | | | - | |  |
| swp1299 | | 0.241 | | | Conserved hypothetical protein | | | - | |  |
| swp0035 | | 0.242 | | | Enoyl-CoA hydratase/isomerase:3-hydroxyacyl-CoA dehydrogenase, | | | - | |  |
| swp0328 | | 0.245 | | | Conserved hypothetical protein | | | - | |  |
| swp4999 | | 0.304 | | | Conserved hypothetical protein | | | - | |  |
| swp4967 | | 0.315 | | | Conserved hypothetical protein | | | - | |  |
| swp3120 | | 0.323 | | | Conserved hypothetical protein | | | - | |  |
| swp0542 | | 0.339 | | | Conserved hypothetical protein | | | - | |  |
| swp1948 | | 0.344 | | | Phosphate acetyltransferase | | | - | |  |
| swp2472 | | 0.359 | | | 2,4-dienoyl-CoA reductase, putative | | | - | |  |
| swp4189 | | 0.362 | | | Conserved hypothetical protein | | | - | |  |
| swp4513 | | 0.373 | | | Porin, Gram-negative type | | | - | |  |
| swp4807 | | 0.376 | | | Hypothetical protein | | | - | |  |
| swp0594 | | 0.437 | | | Hypothetical protein | | | - | |  |
| swp4784 | | 0.451 | | | Conserved hypothetical protein | | | - | |  |
| swp2923 | | 0.453 | | | Cytochrome c3 | | | - | |  |
| swp1442 | | 0.468 | | | NrfJ-related protein | | | - | |  |
| swp0888 | | 0.475 | | | Hypothetical protein | | | - | |  |
| swp3409 | | 0.492 | | | Hypothetical protein | | | - | |  |
| swp1835 | | 2.119 | | | Conserved hypothetical protein | | | - | |  |
| swp2815 | | 2.171 | | | Conserved hypothetical protein | | | - | |  |
| swp4254 | | 2.267 | | | GGDEF domain protein | | | - | |  |
| swp0264 | | 2.431 | | | Transposase, putative | | | - | |  |
| swp0265 | | 3.216 | | | Hypothetical protein | | | - | |  |
| swp3550 | | 3.822 | | | Omega-3 polyunsaturated fatty acid synthase PfaA | | | - | |  |
| swp4362 | | 6.895 | | | Conserved hypothetical protein | | | - | |  |
| swp2323 | | 9.414 | | | Conserved hypothetical protein | | | - | |  |
